# Risk factors for neonatal mortality: an umbrella review of systematic reviews and meta-analyses

**DOI:** 10.1016/j.eclinm.2025.103525

**Published:** 2025-10-06

**Authors:** Bereket Kefale, Jonine Jancey, Amanuel T. Gebremedhin, Daniel Gashaneh Belay, Sylvester Dodzi Nyadanu, Gavin Pereira, Gizachew A. Tessema

**Affiliations:** a Curtin School of Population Health, Curtin University, Perth, Western Australia, Australia; b Department of Reproductive and Family Health, School of Public Health, College of Medicine and Health Sciences, Wollo University, Dessie, Ethiopia; c enAble Institute, Curtin University, Perth, Western Australia, Australia; d School of Nursing and Midwifery, Edith Cowan University, Perth, Western Australia, Australia; e Nutrition and Health Innovation Research Institute, School of Medical and Health Sciences, Edith Cowan University, Perth, Western Australia, Australia; f Department of Epidemiology and Biostatistics, Institute of Public Health, College of Medicine and Health Sciences, University of Gondar, Gondar, Ethiopia; g School of Public Health, University of Adelaide, Adelaide, South Australia, Australia

**Keywords:** Antenatal care, Health facility delivery, Neonatal mortality, Preterm birth, Risk factors, Umbrella review

## Abstract

**Background:**

Neonatal mortality (NM) remains a persistent global public health challenge, necessitating a comprehensive understanding of its associated risk factors to guide effective interventions. Despite multiple systematic reviews and meta-analyses varying in quality, scope, and conclusions, an umbrella review synthesising this evidence was lacking.

**Methods:**

We conducted an umbrella review using six major databases: Embase, Medline, Global Health, CINAHL, Web of Science, and Scopus, along with regional databases, systematic review repositories, and grey literature sources. Eligible studies were systematic reviews and meta-analyses published from 1990 to 22 July 2025 reporting associations between risk factors and all-cause NM based on at least two primary studies. The quality of the reviews was assessed using a measurement tool to assess systematic reviews (AMSTAR-2). Evidence on risk factors was summarised and graded. The review protocol was registered with PROSPERO (CRD42023455542).

**Findings:**

Of 10,610 retrieved records, 64 systematic reviews and meta-analyses were included, five of which were systematic reviews without meta-analyses. We identified 54 unique risk factors across 73 outcome–exposure associations. *Probable evidence* indicated an increased risk of NM associated with maternal age ≥35 years, low occupational status, arsenic exposure, prenatal opioid exposure, maternal overweight and obesity, history of stillbirth, maternal death, severe maternal morbidity, hypertensive disorders of pregnancy, haemorrhagic disorders, anaemia in pregnancy, preterm birth, low birth weight and delayed initiation of breastfeeding (≥24 h). *Probable evidence* also supported the protective effects of antenatal care uptake and health facility delivery. Evidence for most other factors was graded as *limited-suggestive* or *limited and non-conclusive.*

**Interpretation:**

This umbrella review identified *probable evidence* for several modifiable maternal and perinatal risk factors associated with NM, emphasising the critical need to improve access to high-quality antenatal, delivery and neonatal care, prevent preterm birth, promote timely initiation of breastfeeding, and address other identified risk factors. However, further high-quality research is required to strengthen the evidence base on these risk factors.

**Funding:**

None.

**Research in context**

**Evidence before this study**

We systematically searched Medline, Embase, Global Health, Web of Science, and Scopus on December 18, 2024, using the terms (factor∗ OR determinant∗ OR predictor∗ OR associat∗ OR effect∗) AND (newborn∗ OR neonat∗) AND (mortalit∗ OR death∗) AND (umbrella∗ OR overview∗ OR “systematic review of systematic reviews” OR meta-review∗). Although numerous factors have been examined in individual systematic reviews and meta-analyses, we did not identify any umbrella review that synthesised and graded the risk factors associated with neonatal mortality (NM).

**Added value of this study**

To our knowledge, this is the first umbrella review to systematically synthesise, appraise, and grade the available evidence on risk factors for NM. We identified 54 distinct risk factors across 73 exposure–outcome associations. *Probable evidence* supported several modifiable factors, including preterm birth, low birth weight, delayed initiation of breastfeeding, antenatal care uptake, health facility delivery, maternal overweight and obesity, maternal death, and comorbidities such as haemorrhagic disorders, hypertensive disorders of pregnancy, and anaemia. Most other factors were graded as *limited-suggestive* or *limited and non-conclusive*, highlighting the priority areas for future research.

**Implications of all the available evidence**

This umbrella review provides robust evidence for several modifiable maternal and perinatal risk factors for NM, emphasising the critical importance of ensuring access to high-quality antenatal, skilled delivery, and neonatal care. Priority interventions should focus on preventing preterm birth, promoting timely initiation of breastfeeding, and improving maternal health through the prevention and management of comorbidities, including haemorrhagic disorders, hypertensive disorders of pregnancy, and anaemia. Addressing maternal malnutrition, including obesity and overweight, and preventing harmful exposures such as opioid use, cigarette smoking and arsenic exposure during pregnancy are also crucial. Further well-designed prospective studies and meta-analyses that address methodological limitations of the existing evidence are needed to strengthen the evidence base, inform future policies and interventions, and accelerate global efforts to reduce preventable neonatal deaths.

## Introduction

Neonatal mortality (NM), defined as the death of a newborn within the first 28 days of life, remains a persistent global public health challenge.^1^ Despite substantial progress in reducing child mortality, with a 63% global decline in under-five mortality since 1990, neonatal deaths continue to account for nearly half of all under-five deaths. In 2023, an estimated 2.3 million neonatal deaths occurred, contributing to approximately 75 million deaths since 2000.^2^

The burden of NM is unevenly distributed across regions and economic contexts. In 2023, newborns in sub-Saharan Africa (SSA) continued to face the highest mortality risk, with rates 13 times greater than those in Australia and New Zealand (26 vs 2 deaths per 1000 live births, respectively). Similarly, newborns in low-income countries are nine times more likely to die than those in high-income countries (HICs).^2^

The primary causes of NM include preterm birth complications, intrapartum events (such as birth trauma and asphyxia), neonatal infections, and congenital anomalies.^3^ These are influenced by a range of preventable or modifiable risk factors, many of which can be addressed through evidence-based interventions. Despite global and regional commitments, including Sustainable Development Goal (SDG) 3.2, which aims to reduce NM to 12 or fewer deaths per 1000 live births by 2030, progress remains inadequate. If the current trends continue, about 65 countries, predominantly low- and middle-income countries (LMICs), are projected to miss this target.^4^

To accelerate progress and inform targeted health interventions, a comprehensive understanding of risk factors associated with NM is essential.^5^, ^6^, ^7^, ^8^ Although several systematic reviews and meta-analyses have explored these risk factors, the evidence remains fragmented. Many reviews focused on a single exposure,^9^, ^10^, ^11^ or were restricted to specific regions.^7^^,^^12^, ^13^, ^14^ Moreover, variations in methodological quality and inconsistencies in findings hinder the translation of evidence into effective health policies and clinical practices. Our previous umbrella review^15^ synthesised evidence on risk factors for under-five and infant mortality but did not specifically examine NM. Neonatal deaths account for approximately two-thirds of infant and half of under-five mortality, and their determinants and prevention strategies often differ from those for later infant and childhood deaths. To date, no umbrella review has consolidated the substantial body of evidence on risk factors for NM. This review addresses that gap by synthesising findings from existing systematic reviews and meta-analyses to provide targeted guidance for interventions and inform policy and practice.

## Methods

### Study design, search strategy, and selection criteria

We conducted an umbrella review of systematic reviews and meta-analyses. An umbrella review (also called review of reviews or overview of reviews) is a tertiary-level research method that systematically synthesises evidence from multiple systematic reviews and meta-analyses on a specific research question.^16^^,^^17^ This umbrella review followed the preferred reporting items for systematic reviews and meta-analyses (PRISMA) reporting guidelines^18^ and the Joanna Briggs Institute (JBI) Manual for evidence synthesis.^16^ The review protocol was registered with the International Prospective Registry of Systematic Reviews (PROSPERO CRD42023455542).^19^

We systematically searched six major databases: Embase, Medline, Global Health, CINAHL, Web of Science, and Scopus, along with regional databases such as the World Health Organization Global Index Medicus and SciELO. Additionally, systematic review repositories, including Cochrane Database of Systematic Reviews, the JBI Evidence-Based Practice Database (JBI EBP), and Epistemonikos were searched, and grey literature was identified through Google Scholar. The reference lists of included reviews were hand-searched to identify any additional eligible studies not retrieved in the primary search.

The search strategy incorporated medical subject headings (MeSH) and relevant keywords. It included publications from 1990 onwards with no geographic or language restrictions (Supplementary Tables S1–S2). The initial search, conducted on November 6, 2023, formed a part of a broader umbrella review on under-five mortality. Due to the volume of evidence identified, the review was later restructured, resulting in a published umbrella review on under-five and infant mortality^15^ and the present umbrella review on NM. A dedicated search for NM was conducted on December 19, 2024, and updated on July 22, 2025. BK developed the search strategy, which was reviewed and approved by the research team.

We included systematic reviews that met the following eligibility criteria: (1) Population: neonates aged 0–28 days and their mothers; (2) Exposure: risk factors of all-cause NM defined in Supplementary Table S4; (3) Outcome: NM; and (4) Study design: systematic reviews, with or without meta-analyses of quantitative studies. Eligible reviews were required to have a well-defined research question, conduct searches in at least one database, include at least two studies per exposure–outcome association, and either compute effect measures quantifying the association or report the presence of an association between risk factors and NM. Risk factors considered too proximal to NM in the causal pathway, such as birth asphyxia, respiratory distress syndrome, and sepsis, were excluded. Reviews focusing on cause-specific NM or special populations were also excluded. Reviews examining the effects of public health interventions for NM, such as immunisation, vitamin supplementation, nutrition interventions, and other maternal and child health measures, were not considered. Additionally, reviews that were primarily theoretical or opinion-based, as well as narrative, scoping, mapping, and rapid reviews, were excluded.

Records obtained from database searches were managed in EndNote 20, where duplicates were removed before being imported into Covidence for screening. Eligibility was assessed independently by two reviewers (BK and DGB), with disagreements resolved through consultation with a third reviewer (GAT).

### Data extraction and quality appraisal

Data extraction was conducted independently by two reviewers (BK and DGB) using a standardised form adapted from the JBI data extraction template for systematic reviews and meta-analyses.^16^ Information extracted from each review included the first author, year of publication, geographic region, review type, database searching date, number of databases searched, use of grey literature, publication date range of primary studies, review guideline used, protocol registration status, quality assessment, heterogeneity level, publication bias, reported effect estimates such as odds ratios (ORs), relative risks (RRs), and hazard ratios (HRs) with 95% confidence intervals (CIs). The methodological quality of reviews was appraised independently by BK and DGB using a measurement tool to assess systematic reviews (AMSTAR 2), with reviews rated as high, moderate, low, or critically low quality. Any discrepancies were resolved through discussion.^20^

### Data analysis

In line with the JBI Manual for Evidence Synthesis and the Cochrane Handbook's guidance on overviews of reviews,^16^^,^^17^ we applied a descriptive evidence synthesis approach rather than re-analysing primary data. A narrative synthesis, summary tables and figures were used to present the extracted data from the included reviews. The Correct covered area (CCA) algorithm was used to quantify the degree of overlap among the primary studies included in multiple reviews reporting the same risk factors.^21^ Overlap was categorised as slight (≤5%), moderate (6–10%), high (11–15%), and very high (>15%). We graded the direction of association and strength of evidence of included systematic reviews with meta-analyses, based on established grading approaches from previous reviews.^22^, ^23^, ^24^ The direction of associations was classified by consistency across meta-analyses or included primary studies if only one meta-analysis was available. Consistency was defined as *consistent* (≥80%), *less consistent* (60–80%), or *unclear/contradictory* (<60%). Associations were then categorised as *consistent positive (++), less consistent positive (+), less consistent null (0), consistent null (00), less consistent negative (−), consistent negative (−−), or unclear/contradictory (?)* (Supplementary Table S3).^22^ We graded the confidence in the evidence by evaluating the consistency and strength of associations, precision, heterogeneity, biological plausibility, as well as the number, quality, and design of the pooled primary studies. The evidence of each association was then categorised *as convincing (Ce), probable (Pe), limited-suggestive (Ls),* or *limited and non-conclusive (Lnc)* (Supplementary Table S4).^23^^,^^24^ To ensure consistency, RRs were used, with ORs converted where applicable. When the necessary information for conversion was unavailable, ORs were approximated as RRs, assuming that NM is a rare outcome (Supplementary Table S5).^25^ For associations examined in multiple meta-analyses, we reported the range of effect sizes in the main textual summaries, with all available effect estimates reported in supplementary tables.

### Ethics

Not applicable, as this study used data from published literature.

### Role of the funding source

There was no funding source for this study.

## Results

**Fig. 1:**
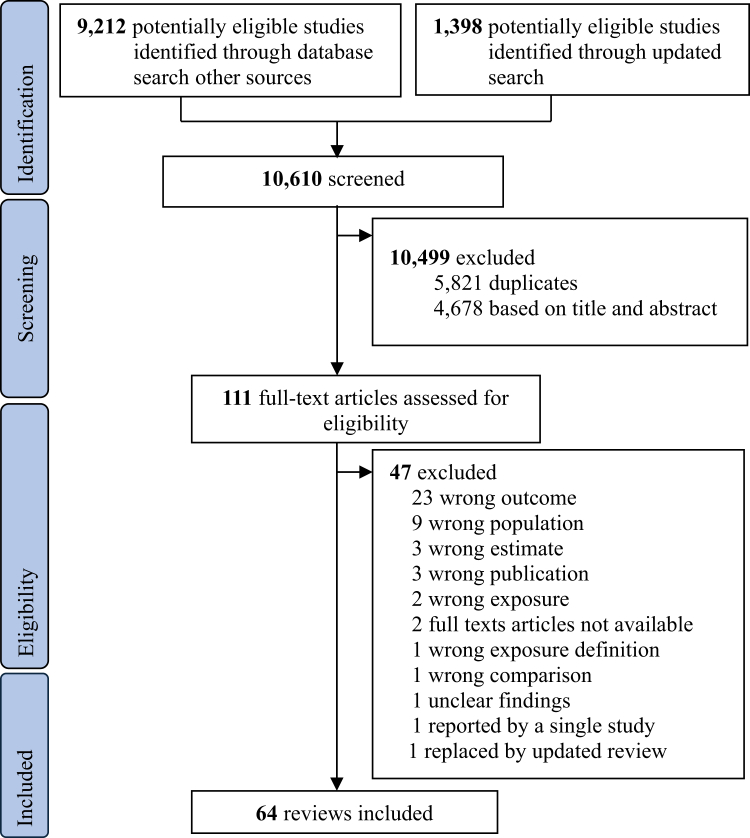
**PRISMA flow diagram**.

A systematic database search identified 10,610 records. After removing 5821 duplicates, 4678 records were excluded following title and abstract screening. A total of 111 full-text articles were assessed for eligibility, of which 64 fulfilled the inclusion criteria and were included in the final synthesis. The remaining 47 articles were excluded for reasons outlined in Supplementary Table S6. Of the included studies, 59 were systematic reviews with meta-analysis and five were systematic reviews without meta-analysis (Fig. 1).

Of the 64 included reviews published between 1998 and 2025, more than three-quarters (76.6%) were published after 2015. Collectively, these reviews included 2742 primary studies, of which over 900, involving more than 160 million participants, specifically focused on risk factors for NM. Forty reviews (62.5%) had a global geographic focus, while 52 (81.3%) reported using at least one reporting guideline, and only twenty (31.2%) registered a pre-specified protocol (Table 1; Supplementary Table S7). The methodological quality of reviews was suboptimal with 23 (35.9%), and 29 (45.3%) rated as low and critically low quality, respectively (Supplementary Figure S1).

**Table 1:** Characteristics of included systematic reviews and meta-analyses (n = 64).

Characteristics	No of reviews
**Year of publication**	
<2000	2 (3.1%)
2000–2015	13 (20.3%)
>2015	49 (76.6%)
**Geographic focus of reviews**	
Global focus	40 (62.5%)
Regional focus	15 (23.4%)
National focus	9 (14.1%)
**Number of primary studies included**	
≤10	4 (6.3%)
11–20	22 (34.4%)
21–30	7 (10.9%)
>30	31 (48.4%)
**Language applied for literature search in the included reviews**	
Only English	29 (45.3%)
Any language	22 (34.4%)
English plus one or more other language	6 (9.4%)
Not reported	7 (10.9%)
**Reporting guidelines used**	
PRISMA	37 (57.8%)
MOOSE	7 (10.9%)
PRISMA and MOOSE	6 (9.4%)
PRISMA and STROBE	1 (1.6%)
NHS CRD	1 (1.6%)
Not reported	12 (18.7%)
**Evidence of pre-specified protocol registration**	
Yes	20 (31.2%)
No	44 (68.8%)
**Quality of reviews**	
High	5 (7.8%)
Moderate	7 (11.0%)
Low	23 (35.9%)
Critically low	29 (45.3%)

MOOSE, Meta-analysis of Observational Studies in Epidemiology; NHS CRD, UK National Health Service Centre for Reviews and Dissemination; PRISMA, Preferred Reporting Items for Systematic Reviews and Meta-Analyses; STROBE, Strengthening the Reporting of Observational Studies in Epidemiology.

We identified 54 distinct risk factors for NM across the included systematic reviews and meta-analyses. These were categorised into six domains: sociodemographic and economic factors (n = 9), environmental factors (n = 4), maternal behavioural and reproductive health-related factors (n = 10), pregnancy-related complications (n = 10), maternal infectious and chronic medical conditions (n = 13), and neonatal factors (n = 8). The most frequently reported factor was antenatal care (ANC) uptake, which was examined in eight reviews, followed by anaemia during pregnancy, interpregnancy interval (IPI), and preterm birth, each reported in four reviews (Table 2; Supplementary Table S8).

**Table 2:** Risk/protective factors of neonatal mortality.

Risk/protective factor	No. of reviews	Primary studies (n)
**Sociodemographic and economic factors**		
Advanced maternal age^7^^,^^26^^,^^27^	3	34
Maternal education^6^^,^^27^	2	302^a^
Paternal education^6^	1	300^b^
Low occupational status^28^	1	3
Poor wealth status^29^^,^^30^	2	6
Aboriginal^31^	1	12
Immigrant^32^	1	14
Single motherhood^27^	1	2
Rural residence^7^^,^^33^	2	10
**Maternal behavioural and reproductive health-related factors**		
Maternal smoking^34^	1	28
Prenatal opioid exposure^35^^,^^36^	2	16
Maternal BMI^37^, ^38^, ^39^	3	33
Interpregnancy weight gain^40^	1	4
Interpregnancy interval^7^, ^8^, ^9^^,^^41^	4	30
Antenatal care uptake^12^, ^13^, ^14^^,^^27^^,^^42^, ^43^, ^44^, ^45^	8	108
Unintended pregnancy^46^	1	5
Health facility delivery^47^^,^^48^	2	38
History of CS delivery^49^	1	5
History of stillbirth^50^	1	7
**Pregnancy-related complications**		
Complication during pregnancy^27^	1	2
Severe maternal morbidity^51^	1	13
Hyperemesis gravidarum^52^	1	5
Mild gestational diabetes mellitus^53^	1	2
Hypertensive disorder of pregnancy^51^^,^^54^	2	38
Haemorrhagic disorder^51^	1	5
Mode of delivery^7^^,^^27^	2	8
Multiple pregnancy^7^	1	2
Placental abruption^55^	1	2
Maternal death^56^	1	4
**Maternal infectious and chronic medical conditions**		
Anaemia during pregnancy^10^^,^^57^, ^58^, ^59^	4	19
Dengue virus infection during pregnancy^60^	1	4
COVID-19 pandemic^61^^,^^62^	2	13
Chlamydia trachomatis^63^	1	3
Hepatitis B infection^5^	1	6
Hepatitis C infections^64^	1	2
HIV infection^65^^,^^66^	2	6
Sub-clinical hypothyroidism^67^	1	6
Psoriasis^68^	1	3
Asthma^69^	1	8
Sickle-cell disease^70^	1	6
Hepatic disorder^51^	1	2
Diabetes^71^	1	19
**Neonatal factors**		
Male neonate^7^^,^^27^	2	6
Low birth weight^7^^,^^27^	2	13
Preterm birth^7^^,^^27^^,^^33^^,^^72^	4	25
Post-term birth^73^	1	5
Low Apgar score^27^	1	3
Late breastfeeding initiation^74^, ^75^, ^76^	3	12
Exclusive breastfeeding^76^	1	2
Birth order^7^	1	4
**Environmental factors**		
Arsenic exposure^11^	1	5
Waste incineration^77^	1	2
Household air pollution (unclean cooking fuels)^78^	1	6
Ambient air pollution^79^	1	7

BMI, body mass index; COVID-19, coronavirus disease 2019; CS, caesarean section.

a The number of primary studies in one review was not clearly reported.

b The number of primary studies for neonatal mortality was not clearly reported.

The identified factors were further classified by modifiability (modifiable vs non-modifiable) and level of influence (individual vs system-level). Most (n = 32) were modifiable individual-level factors, such as maternal smoking, maternal body mass index, prenatal maternal opioid exposure, and IPI. Non-modifiable individual-level factors included advanced maternal age, single motherhood, male neonate, history of caesarean section delivery, history of stillbirth, and multiple pregnancy. Modifiable system-level factors, such as ambient air pollution, waste incineration, arsenic exposure were less frequently reported. Non-modifiable system-level factors included rural residence, aboriginal status, and immigrant status (Supplementary Figure S2).

**Fig. 2:**
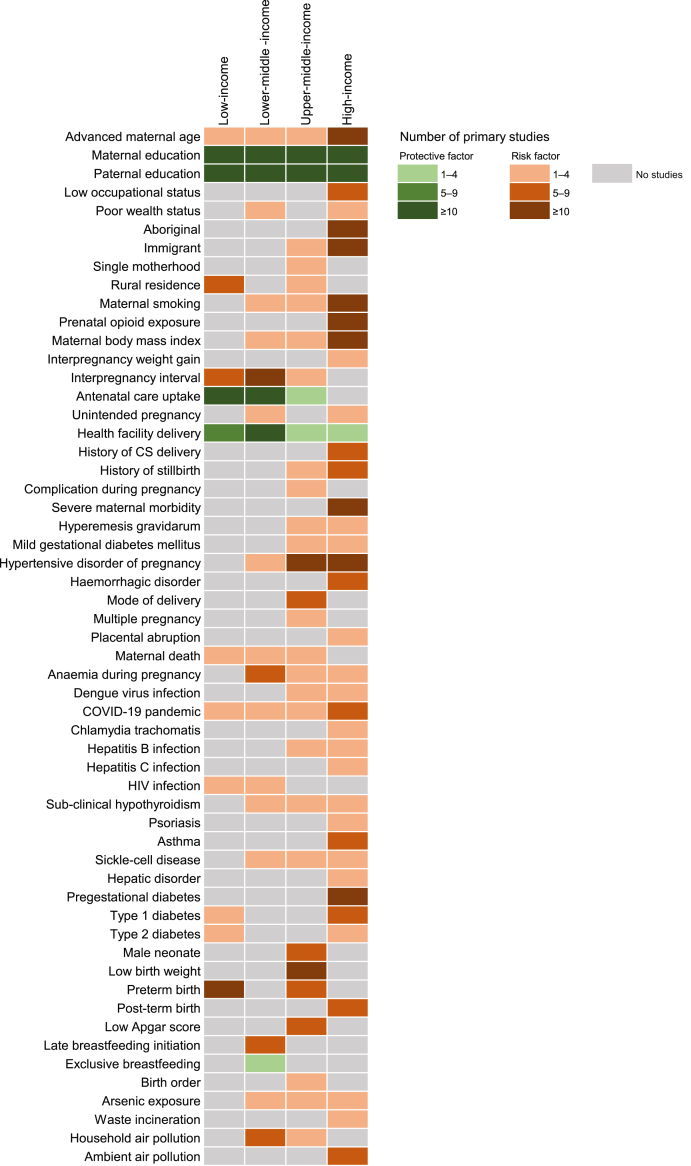
**Evidence on neonatal mortality risk factors across World Bank income groups**. This heatmap shows the number of primary studies reporting associations between specific risk factors and neonatal mortality, stratified by 2024–2025 World Bank country income group: low-, lower-middle-, upper-middle-, and high-income countries.

The evidence on risk factors varied across regions. Higher maternal and paternal education were identified as protective factors across all World Bank income groups. Evidence on advanced maternal age, maternal smoking and prenatal opioid exposure was predominantly originated from HICs, whereas evidence on ANC uptake, health facility delivery, and IPI was mainly originated from low- and lower-middle income countries (Fig. 2).

A total of 73 associations were identified from the included meta-analyses, of which 53 had sufficient data for evidence grading. Among these, 24 were graded as having *probable evidence*, 26 as *limited-suggestive evidence*, and three as *limited and non-conclusive evidence*. In terms of consistency, 23 associations were classified as *consistent positive*, nine as *less consistent positive*, six as *consistent negative*, four as *consistent null*, two as *less consistent null*, and nine as having *unclear or contradictory direction* (Supplementary Table S9).

**Fig. 3:**
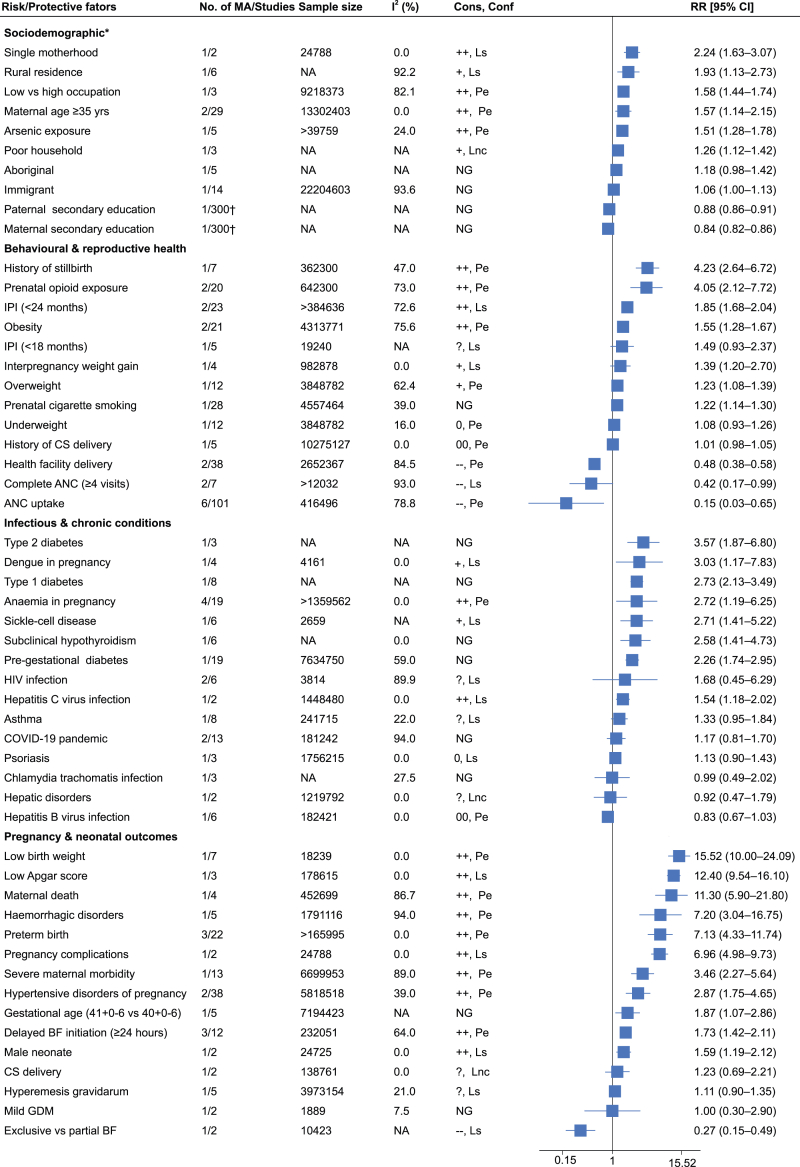
**Forest plot summarising the evidence from meta-analyses on neonatal mortality**. ∗Included economic and environmental factors. †The number of primary studies for the association not clearly reported. +indicates less consistent positive association. ++ indicates consistent positive association. ? indicates unclear or contradictory direction. 0 indicates less consistent null association. 00 indicates consistent null association. −−indicates consistent negative association. ANC, antenatal care; BF, breastfeeding; CI, confidence interval; Cons, consistency of association; Conf, confidence in the evidence; COVID-19, Coronavirus Disease 2019; CS, caesarean section; GDM, gestational diabetes mellitus; I^2^, heterogeneity statistic; IPI, interpregnancy interval; Lnc, limited and non-conclusive evidence; LS, Limited-suggestive evidence; MA, meta-analyses; NA, not available; NG, not graded; Pe, probable evidence; RR, relative risk; yrs, years. For factors with multiple meta-analyses, the RR shown is the largest RR for risk factors and the smallest RR for protective factors.

Nine meta-analyses^6^^,^^11^^,^^26^, ^27^, ^28^, ^29^, ^30^, ^31^, ^32^, ^33^ identified 17 associations between socio-demographic, economic, and environmental factors and NM. Of these, 11 were graded: three as *probable evidence*, seven as *limited-suggestive*, and one as having *limited and non-conclusive evidence*. *Probable evidence was found* for associations between advanced maternal age (≥35 years) (two meta-analyses; 29 studies; n = 13,302,403), with RRs ranging from 1.48 [95% CI 1.30–1.67]^26^ to 1.57 [95% CI 1.14–2.15],^27^ low occupational status (one meta-analysis^28^; six studies; n = 9,218,373 RR 1.58, [95% CI 1.44–1.74]) and arsenic exposure (one meta-analysis^11^; five studies; n > 39,759; RR 1.51 [95% CI 1.28–1.78]) with NM (Fig. 3; Supplementary Table S9).

A total of 18 meta-analyses^8^^,^^9^^,^^12^, ^13^, ^14^^,^^27^^,^^34^^,^^37^, ^38^, ^39^, ^40^, ^41^, ^42^, ^43^^,^^47^, ^48^, ^49^, ^50^ reported 21 associations between maternal behavioural and reproductive health-related factors and NM. Among these, 16 associations were graded: 10 as *probable evidence* and six as having *limited-suggestive evidence*. *Probable evidence* was found for the protective effect of ANC uptake, assessed in six meta-analyses^12^^,^^13^^,^^27^^,^^42^, ^43^, ^44^ including 108 studies (n = 416,496), with RRs ranging from 0.15 [95% CI 0.03–0.65]^27^ to 0.67 [95% CI 0.55–0.80]^43^; and for health facility delivery, investigated in two meta-analyses^47^^,^^48^ including 38 studies (n = 2,652,367), with RRs ranging from [0.71 [95% CI 0.54–0.87]^48^ to 0.48 [95% CI 0.38–0.58].^47^ *Probable evidence* was also identified for associations including prenatal opioid exposure (two meta-analyses^35^^,^^36^; 16 studies; n = 642,300), with RRs ranging from 2.73 [95% CI 1.41–5.28]^36^ to 4.05 [95% CI 2.12–7.72]^35^; maternal obesity (two meta-analyses^37^^,^^38^; 21 studies; n = 4,313,771), with RRs ranging from 1.45 [95% CI 1.29–1.64]^38^ to 1.55 [95% CI 1.28–1.67]^37^; maternal overweight (one meta-analysis^37^; five studies; n = 19,240; RR 1.23 [95% CI 1.08–1.39]); and history of stillbirth (one meta-analysis^50^; nine studies; n = 362,300; RR 4.23 [95% CI 2.64–6.72]) (Fig. 3; Supplementary Table S9).

Eighteen meta-analyses^5^^,^^10^^,^^51^^,^^57^, ^58^, ^59^, ^60^, ^61^, ^62^, ^63^, ^64^, ^65^, ^66^, ^67^, ^68^, ^69^, ^70^, ^71^ examined 15 associations between maternal infectious and chronic medical conditions and NM. Of these, nine were graded: two with *probable evidence*, six with *limited suggestive*, and one with *limited and non-conclusive evidence*. *Probable evidence* was found for anaemia in pregnancy (four meta-analyses^10^^,^^57^, ^58^, ^59^; 19 studies; n = 1,359,562), with RRs ranging from 1.25 [95% CI 1.16–6.25]^59^ to 2.72 [95% CI 1.19–6.25],^10^ and for hepatitis B virus (one meta-analysis^64^; six studies; n = 182,421; RR 0.83 [95% CI 0.67–1.03]) (Fig. 3; Supplementary Table S9).

Eleven meta-analyses^27^^,^^33^^,^^51^^,^^52^^,^^54^^,^^56^^,^^73^, ^74^, ^75^, ^76^^,^^78^ examined 20 associations between pregnancy complications or neonatal factors and NM, with 17 graded for strength and consistency of evidence. Nine associations had *probable evidence*, 7 were *limited-suggestive*, and one had *limited and non-conclusive evidence*. *Probable evidence* was found for maternal death (one meta-analysis^56^; four studies; n = 452,699; RR 11.30 [95% CI 5.90–21.80]), severe maternal morbidity (one meta-analysis^51^; 13 studies; n = 6,699,953; RR 3.46 [95% CI 2.27–5.64]), hypertensive disorders of pregnancy (HDP) (two meta-analyses^51^^,^^54^; 38 studies; n = 5,818,518), with RRs ranging from 1.55 [95% CI 1.18–2.02]^54^ to 2.87 [95% CI 1.75–4.65]^51^, and haemorrhagic disorders (one meta-analysis^51^; five studies; n = 1,791,116; RR 7.20 [95% CI 3.04–16.75]). *Probable evidence* was also identified for preterm birth (three meta-analyses^27^^,^^33^^,^^72^; 22 studies; n > 165,995), with RRs from 1.32 [95% CI 1.07–1.58)^33^ to 7.13 [95% CI 4.33–11.74],^27^ low birth weight (one meta-analysis^27^; Seven studies; n = 18,239; RR 15.52 [95% CI 10.00–24.09]), and delayed breastfeeding initiation (≥24 h) (three meta-analyses^74^, ^75^, ^76^; 12 studies; n = 232,051), with RRs from 1.58 [95% CI 1.26–2.50^74^ to 1.73 [95% CI 1.42–2.11]^75^) (Fig. 3; Supplementary Table S9).

Ninety-four effect estimates from 58 meta-analyses covering 73 associations were identified. Heterogeneity (I^2^) was reported for 68 estimates, with values ranging from 0.0% to 97.0%; of these, 34 exhibited substantial heterogeneity (I^2^ >50%) and 28 demonstrated considerable heterogeneity (I^2^ >75%). I^2^ was unreported for the remaining 25 estimates. Publication bias was reported for 32 estimates: no evidence of bias was found in 26, while six showed evidence of bias (Supplementary Table S9). A total of 11 associations were reported in multiple meta-analyses. The degree of overlap, assessed using CCA, ranged from 0.0% for advanced maternal age to 50.0% for delayed breastfeeding initiation and maternal HIV infection, with a high or very high degree of overlap observed in eight associations (Supplementary Table S10).

## Discussion

This umbrella review is the first to comprehensively and systematically appraise and grade the evidence on risk factors for NM. We synthesised the findings of 64 systematic reviews and meta-analyses, identified 54 unique factors and 73 exposure–outcome associations, of which 53 associations were graded. Twenty-four associations were supported by *probable evidence*, 26 with *limited-suggestive*, and three with *limited and non-conclusive evidence. Probable evidence* indicated increased risks associated with maternal age ≥35 years, low occupational status, arsenic exposure, prenatal opioid exposure, maternal overweight and obesity, history of stillbirth, severe maternal morbidity, HDP, haemorrhagic disorder, maternal death, anaemia in pregnancy, preterm birth, low birth weight, and delayed breastfeeding initiation (≥24 h). *Probable evidence* was also supported protective effects of ANC uptake and health facility delivery. Most other associations were graded as *limited-suggestive* or *limited and non-conclusive*.

Advanced maternal age (≥35 years) was associated with an increased risk of NM.^26^^,^^27^ The evidence was graded as *probable evidence* with *consistent positive associations*. One meta-analysis^26^ showed considerable heterogeneity without exploring potential sources, while the other found no heterogeneity.^27^ Neither meta-analysis assessed publication bias and NM was presented as a secondary outcome in one of these reviews.^26^ The evidence base was mainly derived from the United States, South America, Europe, and Asia, with minimal representation from Africa. Thus, these findings underscore the need for further studies from underrepresented regions, particularly SSA, where three-quarters (75.6%) of global neonatal deaths occur. Low maternal occupation status or social class was also associated with a higher risk of NM, supported by *probable evidence and consistent positive associations*.^28^ Although considerable heterogeneity was reported, no evidence of publication bias was reported. Nevertheless, all included studies were conducted in the United Kingdom and the Republic of Ireland, limiting the generalisability of these findings to other contexts.

Prenatal maternal opioid exposure (any opiate) was identified as a risk for NM.^35^^,^^36^ The evidence was graded as *probable*, with *consistent positive associations* observed. One reported substantial heterogeneity,^35^ while the other did not provide this estimate, and neither assessed the potential for publication bias. Exposure ascertainment varied across studies, with many relying on urine tests and others on maternal self-report, contributing to heterogeneity across studies. Several studies did not adequately adjust for potential confounders, such as co-exposure to tobacco and other substances. The elevated risk reported may, in part, be explained by other factors disproportionately affecting women with opioid use, including sexually transmitted infections, poor nutrition, exposure to violence, poor access to maternal and child health services, and multiple substance use. While one meta-analysis found no association for heroin and methadone use individually, the increased risk associated with ‘any opiate” use likely reflects the cumulative effects of concurrent exposures. These findings warrant cautious interpretation and highlight the need for further investigation to address methodological limitations.

Pre-pregnancy maternal body mass index (BMI) plays a key role in neonatal survival. *Probable evidence* supported the increased risk of NM among overweight women, with *less consistent positive associations.*^37^ Similarly, *probable evidence* indicated an increased risk of neonatal death among obese mothers.^37^^,^^38^ Considerable heterogeneity was observed in one meta-analysis,^37^ and evidence of publication bias was reported in another.^38^ Most included studies relied on self-reported BMI, which may introduce recall and social desirability bias and contribute to heterogeneity. Moreover, the available evidence predominantly originated from HICs, underscoring the need for high-quality studies from underrepresented settings.

The evidence on the association between short IPI and NM remains mixed.^8^^,^^9^^,^^41^ One meta-analysis^41^ examining an IPI of less than 18 months compared with 36–60 months found *limited-suggestive evidence*, with *unclear or contradictory associations,* and confidence intervals crossing the null. Conversely, two other meta-analyses,^8^^,^^9^ showed *limited-suggestive evidence* supporting *consistent positive associations* between short IPIs (<24 months) and NM, with substantial heterogeneity across primary studies. The evidence is largely derived from cross-sectional studies in LMICs, where higher baseline risks related to limited healthcare access, maternal and child malnutrition, and greater disease exposure may exacerbate the effects of short IPIs. These findings may not be generalisable to HICs, where stronger healthcare systems, better maternal care, and improved socio-economic conditions usually mitigate such risks.

*Probable evidence* from one meta-analysis showed *consistent positive associations* between a history of stillbirth and an increased risk of NM. As the evidence was derived exclusively from studies conducted in HICs and upper-middle-income countries further research is warranted in low-and lower-middle-income settings, and along with investigations into the underlying mechanisms for this association.

ANC uptake plays a critical role in reducing NM, supported by *consistent negative associations*.^12^, ^13^, ^14^^,^^27^^,^^42^, ^43^, ^44^, ^45^ *Limited-suggestive evidence* indicated that complete ANC uptake (≥4 visits) was associated with reductions in NM compared to incomplete ANC.^14^^,^^45^ Although these findings reflect outcomes based on the earlier four-visit model, they support the World Health Organization's current recommendation of a minimum of eight ANC contacts, to optimise maternal and neonatal outcomes.^80^ Probable evidence supported the protective effect of any ANC uptake compared to no ANC uptake,^12^^,^^13^^,^^27^^,^^42^, ^43^, ^44^ although considerable heterogeneity was observed across studies within the included meta-analyses. This evidence was drawn exclusively from LMICs, particularly SSA, highlighting the need for data from HICs to improve the generalisability of such findings.

Health facility delivery remains a key factor for reducing NM, supported by *probable evidence* and *consistent negative associations*.^47^^,^^48^ Despite this, considerable heterogeneity and evidence of publication bias were identified across the primary studies included in the meta-analyses. With the exception of one study, all evidence was drawn from LMICs, where home births are commonly attended by traditional birth attendants in settings lacking adequate sanitation, clean delivery surfaces, and essential medical supplies. While further studies from HICs are warranted, these findings provide robust evidence supporting the prioritisation of equitable access to high-quality, facility-based delivery care as a key intervention to reduce preventable neonatal deaths.

Preterm birth is a major risk factor for NM, supported by *probable evidence* and *consistent positive associations*.^27^^,^^33^^,^^72^ One meta-analysis^72^ reported considerable heterogeneity, while another did not assess publication bias.^27^ Similarly, low birth weight was supported by *probable evidence* and *consistent positive associations*. However, all included studies were conducted in Ethiopia and Brazil for preterm birth, and only in Brazil for low birth weight, limiting generalisability of the findings. The results emphasise the need for further research in diverse settings and the implementation of targeted interventions aimed at preventing preterm birth and low birth weight and improving neonatal care for preterm and low birth weight infants.

Delayed initiation of breastfeeding (≥24 h) was associated with an increased risk of NM.^74^, ^75^, ^76^ The evidence was graded as *probable,* with *consistent positive associations*. Furthermore, a meta-analysis indicated an increased risk of NM in neonates initiating breastfeeding at 1–23 h and ≥24 h compared to those initiating within the first hour.^76^ Although this evidence was exclusively derived from LMICs, it underscores the importance of investing in the promotion of early breastfeeding initiation as a critical intervention to improve neonatal and childhood survival.

Maternal death was identified as a risk factor of NM, supported by *probable evidence and consistent positive associations*, although the meta-analyses reported considerable heterogeneity across included studies.^56^ All the evidence originated from LMICs, where 92% of global maternal deaths occur,^81^ often characterised by limited access to quality emergency obstetric and neonatal care services, despite a disproportionately high burden of pregnancy and adverse birth outcomes.

Maternal comorbidities, including severe maternal morbidity,^51^ haemorrhagic disorders,^51^ HDP,^27^^,^^54^ and anaemia in pregnancy,^10^^,^^57^, ^58^, ^59^ were associated with an increased risk of NM, supported by *probable evidence* and *consistent positive associations.* These findings emphasise the importance of strengthening ANC care services, including comprehensive screening, early detection, and timely management of maternal comorbidities, to improve neonatal survival outcomes. These findings also call for further research in LMICs to strengthen the evidence base and inform contextually relevant, evidence-based interventions.

Conversely, *probable evidence* indicated *consistent null associations* for longer IPIs, history of CS delivery, and Hepatitis B virus infection, and a *less consistent null association* for maternal underweight. Evidence for all other factors was graded as limited-suggestive or limited and non-conclusive, due to the small number of studies available and methodological shortcomings.

While no factor achieved the threshold for *convincing evidence*, risk and protective factors supported by *probable evidence* should be prioritised in policy and programming. For factors with *limited-suggestive evidence*, further well-designed research is needed; however, targeted interventions may still be warranted in high-risk settings. Factors with *limited and non-conclusive evidence* reflect substantial uncertainty and are not appropriate for policy action at this stage, highlighting the need for additional high-quality studies to strengthen the evidence base.

To our knowledge, this is the first umbrella review to systematically summarise, appraise, and grade the evidence on risk factors for NM. We conducted an extensive search of six major databases, along with regional databases, systematic review repositories, and grey literature sources, without geographic or language restrictions. Grading approaches were applied to assess the consistency and certainty of evidence on risk factors of NM, to inform evidence-based policy and intervention priorities, and to identify research gaps. However, the findings of this umbrella review should be interpreted in light of the following limitations. Most of the included systematic reviews were rated as low or critically low quality, which undermines overall confidence in the findings. Nevertheless, certain AMSTAR 2 criteria,^20^ such as reporting the funding sources of included primary studies, were not standard practice, including in recent reviews. This likely reflects broader reporting limitations rather than inherent methodological weaknesses. Similarly, many older reviews did not register a protocol, as protocol registration was not widely adopted at the time. While we acknowledge the importance of consistent and standardised reporting, the implications of these limitations should be interpreted in context. These factors may influence quality ratings but do not necessarily compromise the quality of the included reviews.

While most included reviews had a global scope, the underlying evidence on risk factors was predominantly drawn from specific geographic regions or economic settings, Moreover, the findings were not disaggregated by geographic region or World Bank Income classifications, limiting their applicability to diverse settings. Although our review included a figure mapping the evidence for each risk factor by income group, using the World Bank 2024–2025 classification, this figure only reflected the distribution of primary studies. It did not show region-specific pooled estimates, which may differ from the overall pooled estimates. Future systematic reviews and meta-analyses may benefit from stratified analyses to improve global relevance of the evidence and inform context-specific policy and interventions.

Associations lacking pertinent information were not graded, limiting the consistency of the grading process, with more than half of the associations graded as *limited-suggestive and non-conclusive* due to the small number of primary studies and study design limitations. Most of the reported associations with NM came from a single meta-analysis. For those reported by multiple meta-analyses, there was a substantial overlap of primary studies. However, the evidence was graded considering the number and quality of the distinct primary studies.

Some primary studies in the included reviews did not adequately adjust for confounders, which may have introduced uncertainty into the reported pooled estimates. Many of the included meta-analyses, along with their underlying primary studies, treated NM as a secondary outcome. This raises concerns about the adequacy of statistical power to robustly estimate NM-related effects, thereby limiting the strength of causal inferences. Furthermore, publication bias and sensitivity analyses were often limited or unreported for secondary outcomes, further affecting the credibility of the findings.

Substantial heterogeneity was reported in nearly half of the included meta-analyses that provided I^2^ statistics. However, in most of these, the sources of heterogeneity were not further explored, largely due to the limited number of primary studies, which constrained subgroup analyses and meta-regression. The major sources of heterogeneity identified across the meta-analyses included variability in sample sizes,^37^^,^^43^^,^^51^ study designs,^9^^,^^44^^,^^48^^,^^51^ geographical regions,^13^^,^^44^^,^^47^ study settings (e.g., community- vs facility-based),^42^^,^^45^ study periods,^37^ population characteristics,^14^^,^^51^ and exposure definitions.^51^^,^^62^ While I^2^ is widely used to quantify heterogeneity, recent methodological research has raised concerns about its interpretability.^82^ Specifically, I^2^ reflects the proportion of total variation across studies attributable to heterogeneity rather than chance. However, it does not convey whether that variation arises form opposing directions or merely differences in the magnitude. For some factors, such as low occupational status,^28^ and haemorrhagic disorders,^51^ and maternal death,^56^ effect estimates consistently pointed in the same direction despite high I^2^, suggesting that heterogeneity primarily reflected differences in magnitude rather than contradictory findings. Reliance on I^2^ alone may obscure clinically meaningful inconsistency. Given these limitations, the observed heterogeneity across multiple meta-analyses in our review should be interpreted with caution. To enhance interpretability and inform decision-making, future meta-analyses may consider reporting prediction intervals, which provide an estimate of the range within which the true effect is expected to lie. Nevertheless, prediction intervals themselves require cautious application, particularly in meta-analyses including a small number of studies, where statistical uncertainty is amplified.

In two-thirds of the pooled estimates, publication bias was not assessed, primarily due to the small number of studies included in the meta-analyses. However, the absence of significant bias may also reflect the limited statistical power of conventional tests, such as funnel plots, Egger's test, and Begg's test in small-study meta-analyses. These limitations warrant caution interpretation of the findings. For future meta-analyses with a limited number of studies, alternative approaches, such as Doi plots and the Luis Furuya-Kanamori (LFK) index, can be informative for assessing asymmetry and are less sensitive to the number of included studies.^83^ Additionally, one systematic review^7^ provided unclear directions and magnitude of associations for reported risk factors, limiting the ability to draw meaningful conclusions. Although the accessibility and quality of health care resources, such as maternal and child health care units, neonatal intensive care units, essential medical equipment, and skilled workforces, are determinants of neonatal survival,^84^^,^^85^ these health system factors were not reported in the included systematic reviews and meta-analyses. Further research exploring these health system factors is warranted to comprehensively explore complex interplay of the health system determinants influencing neonatal outcomes.

In conclusion, this umbrella review identified *probable evidence* for several modifiable risk factors associated with NM. To address NM, priority interventions should focus on expanding equitable access to high-quality antenatal, skilled delivery and neonatal care. Preventing preterm birth, promoting timely initiation of breastfeeding, and improving maternal health through the prevention and management of comorbidities, such as haemorrhagic disorders, HDP, and anaemia, are essential. Addressing maternal malnutrition, including obesity and overweight, and preventing harmful exposures, such as opioid use, cigarette smoking, and arsenic exposure during pregnancy, are also crucial. Additionally, providing targeted support for families experiencing socioeconomic disadvantage remains vital. Further well-designed prospective studies and meta-analyses that address methodological limitations of the existing evidence, mainly from under-represented regions, such as low- and lower-middle-income countries, are needed to strengthen the evidence base, inform evidence-based policies and interventions, and accelerate global efforts to reduce preventable neonatal deaths.

## Contributors

BK was involved in conceptualisation, data curation, formal analysis, investigation, methodology, project administration, software, visualisation, writing—original draft, and writing—review and editing. JJ contributed to conceptualisation, supervision, validation, writing—review and editing. ATG was involved in conceptualisation, supervision, validation, writing—review and editing. DGB contributed to data curation, formal analysis, investigation, writing—review and editing. SDN was involved in methodology, validation, writing—review and editing. GP contributed to conceptualisation, supervision, validation, writing—review and editing. GAT was involved in conceptualisation, supervision, validation, writing—review and editing. BK, ATG, GP, and GAT had full access to and verified the data. All authors have read and approved the final manuscript, and BK, JJ, ATG, GP, and GAT take the responsibility for the decision to submit.

## Data sharing statement

All data used in this study were extracted from publicly available systematic reviews. The tables and figures generated in this study are included within the manuscript and supplementary materials.

## Declaration of interests

All authors declare that they have no conflicts of interest.

## Appendix A Supplementary data

Revised Supplementary File
