# Supplementary material for: Risk factors for neonatal mortality: an umbrella review of systematic reviews and meta-analyses
Source: eClinicalMedicine. 2025 Oct 6;88:103525. doi: 10.1016/j.eclinm.2025.103525 (PMC12572802; doi:10.1016/j.eclinm.2025.103525)
Supplement: Revised Supplementary File [file mmc1.docx]

**Risk factors for neonatal mortality: an umbrella review of Systematic reviews and meta-analyses**

**Authors:** Bereket Kefale, Jonine Jancey, Amanuel T Gebremedhin, Daniel G Belay, Sylvester Dodzi Nyadanu, Gavin Pereira, Gizachew A Tessema

**Table of Contents**

[Supplementary Table S1: Search strategy for the umbrella review on risk factors of neonatal mortality 2](#_Toc207915889)

[Supplementary Table S2: Updated search strategy for the umbrella review on risk factors of neonatal mortality 5](#_Toc207915890)

[Supplementary Table S3: Direction of association grading criteria 9](#_Toc207915891)

[Supplementary Table S4: Strength of evidence grading criteria 10](#_Toc207915892)

[Supplementary Table S5: Formula used for converting odds ratio (OR) to relative risk (RR) 11](#_Toc207915893)

[Supplementary Table S6: Excluded reviews and reasons for exclusion after full-text review 12](#_Toc207915894)

[Supplementary Table S7: Characteristics of systematic reviews and meta-analyses 14](#_Toc207915895)

[Supplementary Table S8: Summary results from systematic reviews and meta-analyses 22](#_Toc207915896)

[Supplementary Table S9: Summary of evidence of meta-analyses on neonatal mortality 27](#_Toc207915897)

[Supplementary Table S10: Study overlaps in the included meta-analyses 31](#_Toc207915898)

[Supplementary Table S11: Calculated relative risks 32](#_Toc207915899)

[Supplementary Figure S1: Quality assessment using AMSTAR 2 tool 35](#_Toc207915900)

[Supplementary Figure S2: Risk factors of neonatal mortality based on modifiability and level of influence 36](#_Toc207915901)

[Supplementary Figure S3: Publication date ranges of systematic reviews 37](#_Toc207915902)

[References 38](#_Toc207915903)

# **Supplementary Table S1: Search strategy for the umbrella review on risk factors of neonatal mortality**

|  | **Concept 1** | **Concept 2** | **Concept 3** |
| --- | --- | --- | --- |
|  | **Risk factors** | **Neonatal mortality** | **Systematic review and meta-analysis** |
| **Keywords**  **(Ovid)** | ( "risk factor*" or "associated factor*" or "factor* associated" or determinant* or predictor* or factor* or effect* or associat*).ti,ab. | ((perinatal or newborn* or new-born* or neonat*) ADJ2 (mortalit* or death*)).ti,ab. | ("systematic review*"or meta-analy* or metaanaly*).ti,ab. |
| **Embase headings** | exp risk factor/ | exp perinatal mortality/ or exp perinatal death/ or exp newborn mortality/ or exp newborn death/ | exp "systematic review"/ or exp meta analysis/ or exp "systematic review (topic)"/ or exp "meta analysis (topic)"/ |
| **Medline headings** | exp Risk Factors/ | exp Perinatal Death/ | exp "systematic review"/ or exp meta-analysis/ or exp "Systematic Reviews as Topic"/ or exp "Meta-Analysis as Topic"/ |
| **Keywords (EBSCO)** | TI ("risk factor*" or "associated factor*" or "factor* associated" or determinant* or predictor* or factor* or effect* or associat*) or AB ("risk factor*" or "associated factor*" or "factor* associated" or determinant* or predictor* or factor* or effect* or associat*) | TI ((perinatal or newborn* or new-born* or neonat*) N2 (mortalit* or death*)) OR AB ((perinatal or newborn* or new-born* or neonat*) N2 (mortalit* or death*)) | TI (systematic review* or meta-analy*) or AB (systematic review* or meta-analy*) |
| **CINAHL headings** | (MH "Risk Factors+") | (MH "Perinatal Death") | (MH "Systematic Review") OR (MH "Meta Analysis") |
| **Keywords**  **Scopus/Web of science** | ("risk factor*" or "associated factor*" or "factor* associated" or determinant* or predictor* or factor* or effect* or associat*) | ("perinatal death*" or "newborn* death*" or "new-born* death*" or "neonat* death*" or "perinatal mortalit*" or "newborn* mortalit*" or "new-born* mortalit*" or "neonat* mortalit*" or "infant mortalit*") | (systematic review* or meta-analy* or metaanaly*) |

**Search strategies for each database**

| **Databases** | **s.no** | **Headings, key terms with proximity operators** |
| --- | --- | --- |
| **Embase**  **(Ovid)** | #1 | exp risk factor/ |
|  | #2 | ("risk factor*" or "associated factor*" or "factor* associated" or determinant* or predictor* or factor* or effect* or associat*).ti,ab. |
|  | #3 | #1 or #2 |
|  | #4 | exp perinatal mortality/ or exp perinatal death/ or exp newborn death/ or exp newborn mortality/ |
|  | #5 | ((perinatal or newborn* or new-born* or neonat*) ADJ2 (mortalit* or death*)).ti,ab. |
|  | #6 | #4 or #5 |
|  | #7 | exp "systematic review"/ or exp meta analysis/ or exp "systematic review (topic)"/ or exp "meta analysis (topic)"/ |
|  | #8 | ("systematic review*"or meta-analy* or metaanaly*).ti,ab. |
|  | #9 | #7 or #8 |
|  | #10 | #3 and #6 and #9 |
|  | #11 | limit (English language and yr="1990 -Current") and (human) and (article or article in press or "preprint) |
| **Medline**  **(Ovid)** | #1 | exp risk factors/ |
|  | #2 | ("risk factor*" or "associated factor*" or "factor* associated" or determinant* or predictor* or factor* or effect* or associat*).ti,ab. |
|  | #3 | #1 or #2 |
|  | #4 | exp Perinatal Death/ |
|  | #5 | ((perinatal or newborn* or new-born* or neonat*) ADJ2 (mortalit* or death*)).ti,ab. |
|  | #6 | #4 or #5 |
|  | #7 | exp "systematic review"/ or exp meta-analysis/ or exp "Systematic Reviews as Topic"/ or exp "Meta-Analysis as Topic"/ |
|  | #8 | ("systematic review*"or meta-analy* or metaanaly*).ti,ab. |
|  | #9 | #7 or #8 |
|  | #10 | #3 and #6 and #9 |
|  | #11 | Limit (English language and yr="1990 -Current") and (human) and (journal article or "preprint) |
| **Global Health**  **(Ovid)** | #1 | exp risk factors/ |
|  | #2 | ("risk factor*" or "associated factor*" or "factor* associated" or determinant* or predictor* or factor* or effect* or associat*).ti,ab. |
|  | #3 | #1 or #2 |
|  | #4 | exp perinatal mortality/ or exp neonatal mortality/ |
|  | #5 | ((perinatal or newborn* or new-born* or neonat*) ADJ2 (mortalit* or death*)).ti,ab. |
|  | #6 | #4 or #5 |
|  | #7 | exp systematic reviews/ or exp meta-analysis/ |
|  | #8 | ("systematic review*"or meta-analy* or metaanaly*).ti,ab. |
|  | #9 | #7 or #8 |
|  | #10 | #3 and #6 and #9 |
|  | #11 | Limit (English language and yr="1990 -Current" and journal article or preprint or theis) |
| **Scopus** | #1 | TITLE-ABS-KEY ("risk factor*" or "associated factor*" or "factor* associated" or determinant* or predictor* or factor* or effect* or associat*) |
|  | #2 | TITLE-ABS-KEY (“perinatal death*" OR "newborn* death*" OR "new-born* death*" OR "neonat* death*" OR "perinatal mortalit*" OR "newborn* mortalit*" OR "new-born* mortalit*" OR "neonat* mortalit*") |
|  | #3 | TITLE-ABS-KEY ("systematic review*" or meta-analy* or metaanaly*) |
|  | #4 | #1 and #2 and #3 |
|  | #5 | Pubyear > 1990 and pubyear < 2024 and (limit-to (language, "English”) and document type- article |
| **Web of science** | #1 | TI=(("risk factor*" or "associated factor*" or "factor* associated" or determinant* or predictor* or factor* or effect* or associat*)) OR AB=(("risk factor*" or "associated factor*" or "factor* associated" or determinant* or predictor* or factor* or effect* or associat*)) |
|  | #2 | TI=(("perinatal death*" or "newborn* death*" or "new-born* death*" or "neonat* death*" or "perinatal mortalit*" or "newborn* mortalit*" or "new-born* mortalit*" or "neonat* mortalit*")) OR AB=(("perinatal death*" or "new-born* death*" or "neonat* death*" or "perinatal mortalit*" or "newborn* mortalit*" or "new-born* mortalit*" or "neonat* mortalit*")) |
|  | #3 | TI=(("systematic review*" or meta-analy* or metaanaly*)) OR  AB=(("systematic review*" or meta-analy* or metaanaly*)) |
|  | #4 | #1 and #2 and #3 |
|  | #5 | Limited to English, review article and article |
| **CINAHL (EBSCO)** | #1 | (MH "Risk Factors+") |
|  | #2 | TI ("risk factor*" or "associated factor*" or "factor* associated" or determinant* or predictor* or factor* or effect* or associat*) or AB ("risk factor*" or "associated factor*" or "factor* associated" or determinant* or predictor* or factor* or effect* or associat*) |
|  | #3 | #1 or #2 |
|  | #4 | (MH "Perinatal Death") |
|  | #5 | TI ((perinatal or newborn* or new-born* or neonat*) N2 (mortalit* or death*)) OR AB ((perinatal or newborn or new-born* or neonat*) N2 (mortalit* or death*)) |
|  | #6 | #4 or #5 |
|  | #7 | (MH "Systematic Review") OR (MH "Meta Analysis") |
|  | #8 | TI (systematic review* or meta-analy* or metanaly*) or AB (systematic review* or meta-analy* or meta-analy*) |
|  | #9 | #7 or #8 |
|  | #10 | #3 and #6 and #9 |
|  | #11 | Limiters - Published Date: 1990-2023, and Language: - English |
| [**Cochrane Database of Systematic Reviews**](https://www.cochranelibrary.com/) | #1 | ("risk factor" or "associated factor" or "factor associated" or determinant or predictor or factor or effect or association):ti,ab,kw - (Word variations have been searched) |
|  | #2 | ("perinatal death" OR "newborn death" OR "new-born death" OR "neonatal death" OR "perinatal mortality" OR "newborn mortality" OR "new-born mortality" OR "neonatal mortality") ti,ab,kw - (Word variations have been searched) |
|  | #3 | #1 and #2 |
|  | #4 | Filter: year: since 1990, Cochrane review |
| **Joanna Briggs Institute** **EBP Database (Ovid)** | #1 | ("risk factor*" or "associated factor*" or "factor* associated" or "associated risk factor*" or determinant* or predictor* or factor* or effect* or associat*).ti,ab. |
|  | #2 | ((perinatal or newborn* or new-born* or neonat*) ADJ2 (mortalit* or death*)).ti,ab. |
|  | #3 | #1 and #2 |
| **Epistemonikos** | #1 | (title:((title:("risk factor*" OR "associated factor*" OR "factor* associated" OR determinant* OR predictor* OR factor* OR effect* OR associate*) OR abstract:("risk factor*" OR "associated factor*" OR "factor* associated" OR determinant* OR predictor* OR factor* OR effect* OR associate*))) OR abstract:((title:("risk factor*" OR "associated factor*" OR "factor* associated" OR determinant* OR predictor* OR factor* OR effect* OR associate*) OR abstract:("risk factor*" OR "associated factor*" OR "factor* associated" OR determinant* OR predictor* OR factor* OR effect* OR associate*)))) |
|  | #2 | (title:((title:("Perinatal death*" OR "perinatal mortalit*" OR "newborn* death*" OR "new-born* death*" OR "newborn* mortalit*" OR "new-born* mortalit*" OR "neonat* death*" OR "neonat* mortalit*") OR abstract:("Perinatal death*" OR "perinatal mortalit*" OR "newborn* death*" OR "new-born* death*" OR "newborn* mortalit*" OR "new-born* mortalit*" OR "neonat* death*" OR "neonat* mortalit*") OR abstract:("Perinatal death*" OR "perinatal mortalit*" OR "newborn* death*" OR "new-born* death*" OR "newborn* mortalit*" OR "new-born* mortalit*" OR "neonat* death*" OR "neonat* mortalit*") OR abstract:("Perinatal death*" OR "perinatal mortalit*" OR "newborn* death*" OR "newborn* mortalit*" OR "neonat* death*" OR "neonat* mortalit*")))) |
|  | #3 | #1 and #2 |
|  | #4 | Publication date since 1990, Publication type: Systematic review |
| **Google**  **Scholar**  (first 200 hits where available) |  | ("risk factors"\|"associated factors"\|determinants\|predictors) ("perinatal mortality"\|"neonatal mortality"\|"newbornl mortality"\|"new-born mortality") ("systematic review"\| meta-analysis\|metaanalysis) |

# **Supplementary Table S2: Updated search strategy for the umbrella review on risk factors of neonatal mortality**

| **Databases** | **s.no** | **Headings, key terms with proximity operators** |
| --- | --- | --- |
| **Embase**  **(Ovid)** | #1 | exp risk factor/ |
|  | #2 | ("risk factor*" or "associated factor*" or "factor* associated" or determinant* or predictor* or factor* or effect* or associat*).ti,ab. |
|  | #3 | #1 or #2 |
|  | #4 | exp perinatal mortality/ or exp perinatal death/ or exp newborn death/ or exp newborn mortality/ |
|  | #5 | ((perinatal or newborn* or new-born* or neonat*) ADJ2 (mortalit* or death*)).ti,ab. |
|  | #6 | #4 or #5 |
|  | #7 | exp "systematic review"/ or exp meta analysis/ or exp "systematic review (topic)"/ or exp "meta analysis (topic)"/ |
|  | #8 | ("systematic review*"or meta-analy* or metaanaly*).ti,ab. |
|  | #9 | #7 or #8 |
|  | #10 | #3 and #6 and #9 |
|  | #11 | limit 10 to (human and english language and yr="2024 -Current" and (article or article in press or "preprint (unpublished, non-peer reviewed)")) |
|  | #12 | limit 10 to (human and (yr="1990 -Current") and (article or article in press or "preprint (unpublished, non-peer reviewed)") and (afrikaans or albanian or arabic or armenian or azerbaidzhani or basque or belorussian or bengali or bulgarian or burmese or bosnian or catalan or chinese or croatian or czech or danish or dutch or esperanto or estonian or finnish or french or gallegan or georgian or german or greek or hebrew or hindi or hungarian or icelandic or indonesian or "irish gaelic" or italian or japanese or korean or latvian or lithuanian or macedonian or malay or maori or mongolian or norwegian or persian or polish or polyglot or portuguese or pushto or romanian or russian or "scottish gaelic" or serbian or sinhalese or slovak or slovene or spanish or swedish or tagalog or thai or turkish or ukrainian or urdu or uzbek or vietnamese)) |
| **Medline**  **(Ovid)** | #1 | exp risk factors/ |
|  | #2 | ("risk factor*" or "associated factor*" or "factor* associated" or determinant* or predictor* or factor* or effect* or associat*).ti,ab. |
|  | #3 | #1 or #2 |
|  | #4 | exp Perinatal Death/ |
|  | #5 | ((perinatal or newborn* or new-born* or neonat*) ADJ2 (mortalit* or death*)).ti,ab. |
|  | #6 | #4 or #5 |
|  | #7 | exp "systematic review"/ or exp meta-analysis/ or exp "Systematic Reviews as Topic"/ or exp "Meta-Analysis as Topic"/ |
|  | #8 | ("systematic review*"or meta-analy* or metaanaly*).ti,ab. |
|  | #9 | #7 or #8 |
|  | #10 | #3 and #6 and #9 |
|  | #11 | limit 10 to (english language and humans and yr="2024 -Current" and english and (journal article or preprint)) |
|  | #12 | limit 10 to (humans and yr="1990 -Current" and (afrikaans or albanian or arabic or armenian or azerbaijani or belorussian or bengali or bosnian or bulgarian or burmese or catalan or chinese or croatian or czech or danish or dutch or esperanto or estonian or finnish or flemish or french or gaelic, scots or georgian or german or greek or hausa or hebrew or hindi or hungarian or icelandic or indonesian or interlingua or italian or japanese or kirghiz or korean or latin or latvian or lithuanian or macedonian or malay or marathi or masai or multilingual or norwegian or persian or polish or portuguese or pushto or rumanian or russian or serbian or slovak or slovene or spanish or swahili or swedish or tagalog or tamil or telugu or thai or turkish or ukrainian or undetermined or urdu or vietnamese or welsh) and (journal article or preprint)) |
| **Global Health**  **(Ovid)** | #1 | exp risk factors/ |
|  | #2 | ("risk factor*" or "associated factor*" or "factor* associated" or determinant* or predictor* or factor* or effect* or associat*).ti,ab. |
|  | #3 | #1 or #2 |
|  | #4 | exp perinatal mortality/ or exp neonatal mortality/ |
|  | #5 | ((perinatal or newborn* or new-born* or neonat*) ADJ2 (mortalit* or death*)).ti,ab. |
|  | #6 | #4 or #5 |
|  | #7 | exp systematic reviews/ or exp meta-analysis/ |
|  | #8 | ("systematic review*"or meta-analy* or metaanaly*).ti,ab. |
|  | #9 | #7 or #8 |
|  | #10 | #3 and #6 and #9 |
|  | #11 | limit 10 to (english and (journal article or preprint or thesis) and yr="2024 -Current") |
|  | #12 | limit 10 to ((arabic or armenian or assamese or azerbaijani or basque or belarusian or bengali or bosnian or breton or bulgarian or burmese or cambodian or catalan or central khmer or chichewa or chinese or croatian or czech or danish or dutch or esperanto or estonian or ewe or filipino or finnish or french or frisian or galician or ganda or georgian or german or greek or haitian or hausa or hebrew or hindi or hungarian or icelandic or igbo or indian or indonesian or interlingua or italian or japanese or kannada or kanuri or karakalpak or kazakh or kinyarwanda or kirgiz or kirundi or korean or lao or laothian or latin or latvian or lingala or lithuanian or luo or macedonian or malagasy or malay or malayalam or maltese or maori or moldavian or mongolian or nepali or nkore or norwegian or "not specified" or palauan or persian or polish or portuguese or romanian or romansh or rundi or russian or samoan or serbian or serbo croatian or setswanese or shona or sinhalese or slovakian or slovenian or somali or spanish or stellingwerf or sudanese or swahili or swedish or tagalog or tajik or tamil or tawahka or thai or tibetan or tigrinya or tiv or tswana or turkish or turkmenian or ukrainian or urdu or uzbek or vietnamese or welsh or western frisian or white russian or xhosa or yiddish or yoruba or zulu) and (journal article or preprint or thesis) and yr="1990 -Current") |
| **Scopus** | #1 | TITLE-ABS-KEY ("risk factor*" or "associated factor*" or "factor* associated" or determinant* or predictor* or factor* or effect* or associat*) |
|  | #2 | TITLE-ABS-KEY (“perinatal death*" OR "newborn* death*" OR "new-born* death*" OR "neonat* death*" OR "perinatal mortalit*" OR "newborn* mortalit*" OR "new-born* mortalit*" OR "neonat* mortalit*") |
|  | #3 | TITLE-ABS-KEY ("systematic review*" or meta-analy* or metaanaly*) |
|  | #4 | #1 and #2 and #3 |
|  | #5 | PUBYEAR > 2023 AND PUBYEAR < 2026 AND ( LIMIT-TO ( DOCTYPE , "ar" ) OR LIMIT-TO ( DOCTYPE , "re" ) ) AND ( LIMIT-TO ( LANGUAGE , "English" ) ) |
|  | #6 | Pubyear > 1990 and pubyear < 2024 and ( TITLE-ABS-KEY ( "risk factor*" or "associated factor*" or "factor* associated" or determinant* or predictor* or factor* or effect* or associat* ) ) AND ( TITLE-ABS-KEY ( "perinatal death*" OR "newborn* death*" OR "new-born* death*" OR "neonat* death*" OR "perinatal mortalit*" OR "newborn* mortalit*" OR "new-born* mortalit*" OR "neonat* mortalit*" ) ) AND ( TITLE-ABS-KEY ( "systematic review*" or meta-analy* or metaanaly* ) ) AND ( LIMIT-TO ( LANGUAGE , "Spanish" ) OR LIMIT-TO ( LANGUAGE , "French" ) OR LIMIT-TO ( LANGUAGE , "German" ) OR LIMIT-TO ( LANGUAGE , "Chinese" ) OR LIMIT-TO ( LANGUAGE , "Russian" ) OR LIMIT-TO ( LANGUAGE , "Portuguese" ) OR LIMIT-TO ( LANGUAGE , "Persian" ) OR LIMIT-TO ( LANGUAGE , "Italian" ) OR LIMIT-TO ( LANGUAGE , "Dutch" ) OR LIMIT-TO ( LANGUAGE , "Danish" ) OR LIMIT-TO ( LANGUAGE , "Korean" ) OR LIMIT-TO ( LANGUAGE , "Slovenian" ) OR LIMIT-TO ( LANGUAGE , "Turkish" ) OR LIMIT-TO ( LANGUAGE , "Ukrainian" ) ) |
| **Web of science** | #1 | TI=(("risk factor*" or "associated factor*" or "factor* associated" or determinant* or predictor* or factor* or effect* or associat*)) OR AB=(("risk factor*" or "associated factor*" or "factor* associated" or determinant* or predictor* or factor* or effect* or associat*)) |
|  | #2 | TI=(("perinatal death*" or "newborn* death*" or "new-born* death*" or "neonat* death*" or "perinatal mortalit*" or "newborn* mortalit*" or "new-born* mortalit*" or "neonat* mortalit*")) OR AB=(("perinatal death*" or "new-born* death*" or "neonat* death*" or "perinatal mortalit*" or "newborn* mortalit*" or "new-born* mortalit*" or "neonat* mortalit*")) |
|  | #3 | TI=(("systematic review*" or meta-analy* or metaanaly*)) OR  AB=(("systematic review*" or meta-analy* or metaanaly*)) |
|  | #4 | #1 and #2 and #3 |
|  | #5 | Limited to 2025 or 2024 (Publication Years) and English (Languages) and Article or Review Article (Document Types) |
|  | #6 | Limited to  Article or Review Article (Document Types) and 2025 or 2024 or 2023 or 2022 or 2021 or 2020 or 2019 or 2018 or 2017 or 2016 or 2015 or 2014 or 2013 or 2012 or 2011 or 2010 or 2009 or 2008 or 2007 or 2006 or 2005 or 2004 or 2003 or 2002 or 2001 or 2000 or 1999 or 1998 or 1997 or 1996 or 1995 or 1994 or 1993 or 1991 or 1990 (Publication Years) and French or German or French or German or Spanish or Italian or Portuguese (Languages) |
| **CINAHL (EBSCO)** | #1 | (MH "Risk Factors+") |
|  | #2 | TI ("risk factor*" or "associated factor*" or "factor* associated" or determinant* or predictor* or factor* or effect* or associat*) or AB ("risk factor*" or "associated factor*" or "factor* associated" or determinant* or predictor* or factor* or effect* or associat*) |
|  | #3 | #1 or #2 |
|  | #4 | (MH "Perinatal Death") |
|  | #5 | TI ((perinatal or newborn* or new-born* or neonat*) N2 (mortalit* or death*)) OR AB ((perinatal or newborn or new-born* or neonat*) N2 (mortalit* or death*)) |
|  | #6 | #4 or #5 |
|  | #7 | (MH "Systematic Review") OR (MH "Meta Analysis") |
|  | #8 | TI (systematic review* or meta-analy* or metanaly*) or AB (systematic review* or meta-analy* or meta-analy*) |
|  | #9 | #7 or #8 |
|  | #10 | #3 and #6 and #9 |
|  | #11 | Limiters – Date: Date: 1990-19/12/2024, Language: - English |
|  | #12 | Limiters – Date: Date: 2024-22/07/2025, Language: - English |
|  | #13 | Limiters – Published, Date: 1990-22/07/2025, and Language: - Spanish, Portuguese, Persian, Arabic, French, and modern Greek |
| [**Cochrane Database of Systematic Reviews**](https://www.cochranelibrary.com/) | #1 | ("risk factor" or "associated factor" or "factor associated" or determinant or predictor or factor or effect or association):ti,ab,kw - (Word variations have been searched) |
|  | #2 | ("perinatal death" OR "newborn death" OR "new-born death" OR "neonatal death" OR "perinatal mortality" OR "newborn mortality" OR "new-born mortality" OR "neonatal mortality") ti,ab,kw - (Word variations have been searched) |
|  | #3 | #1 and #2 |
|  | #4 | Filter: year: since 2024 |
| **Joanna Briggs Institute EBP Database (Ovid)** | #1 | ("risk factor*" or "associated factor*" or "factor* associated" or "associated risk factor*" or determinant* or predictor* or factor* or effect* or associat*).ti,ab. |
|  | #2 | ((perinatal or newborn* or new-born* or neonat*) ADJ2 (mortalit* or death*)).ti,ab. |
|  | #3 | #1 and #2 |
|  | #4 | limit 3 to yr="2024 -Current" |
| **Epistemonikos** | #1 | (title:((title:("risk factor*" OR "associated factor*" OR "factor* associated" OR determinant* OR predictor* OR factor* OR effect* OR associate*) OR abstract:("risk factor*" OR "associated factor*" OR "factor* associated" OR determinant* OR predictor* OR factor* OR effect* OR associate*))) OR abstract:((title:("risk factor*" OR "associated factor*" OR "factor* associated" OR determinant* OR predictor* OR factor* OR effect* OR associate*) OR abstract:("risk factor*" OR "associated factor*" OR "factor* associated" OR determinant* OR predictor* OR factor* OR effect* OR associate*)))) |
|  | #2 | (title:((title:("Perinatal death*" OR "perinatal mortalit*" OR "newborn* death*" OR "new-born* death*" OR "newborn* mortalit*" OR "new-born* mortalit*" OR "neonat* death*" OR "neonat* mortalit*") OR abstract:("Perinatal death*" OR "perinatal mortalit*" OR "newborn* death*" OR "new-born* death*" OR "newborn* mortalit*" OR "new-born* mortalit*" OR "neonat* death*" OR "neonat* mortalit*") OR abstract:("Perinatal death*" OR "perinatal mortalit*" OR "newborn* death*" OR "new-born* death*" OR "newborn* mortalit*" OR "new-born* mortalit*" OR "neonat* death*" OR "neonat* mortalit*") OR abstract:("Perinatal death*" OR "perinatal mortalit*" OR "newborn* death*" OR "newborn* mortalit*" OR "neonat* death*" OR "neonat* mortalit*")))) |
|  | #3 | #1 and #2 |
|  | #4 | Publication date since 2024, Publication type: Systematic review |
| SciELO | #1 | (*"risk factor" OR "associated factor" OR "factor associated" OR determinant OR predictor OR factor OR effect OR associat) AND ("perinatal death" OR "newborn death" OR "new-born death" OR "neonatal death" OR "perinatal mortality" OR "newborn mortality" OR "new-born mortality" OR "neonatal mortality") AND ("systematic review" OR "meta-analysis" OR "meta-analyses" OR "metaanalysis" OR "metaanalyses") |
| WHO Global Index Medicus | #1 | ("risk factor*" OR "associated factor*" OR "factor* associated" OR determinant* OR predictor* OR factor* OR effect* OR associat*) AND ("perinatal death*" OR "newborn* death*" OR "new-born* death*" OR "neonat* death*" OR "perinatal mortalit*" OR "newborn* mortalit*" OR "new-born* mortalit*" OR "neonat* mortalit*") AND ("systematic review*" OR meta-analy* OR metaanaly*) |
| **Google**  **Scholar**  (first 200 hits where available) |  | ("risk factors"\|"associated factors"\|determinants\|predictors) ("perinatal mortality"\|"neonatal mortality"\|"newborn mortality"\|"newborn mortality") ("systematic review"\| meta-analysis\|meta analysis) |

# **Supplementary Table S3: Direction of association grading criteria**

| **Category on importance of risk factors***^1^* | **Grading criteria** |
| --- | --- |
| “++”  (Consistent positive association) | A variable shows a significant positive association or reports effect sizes (RR) greater than or equals to.5 in at least 80% of reviews. If only a single review is available, the variable shows a significant positive association or effect sizes greater than and equals to 1.5 in at least 80% of the included primary studies. |
| “+”  (Less consistent positive association) | A variable shows a significant positive association or reports effect sizes (RR) greater than 1.5 in at least 60% of reviews. If only a single review is available, the variable shows a significant positive association or effect sizes greater than or equals to 1.5 in at least 60% of the included primary studies. |
| “00”  (Consistent null association) | The variable shows a null association in at least 80% of reviews. It also means that either a single review included the variable and demonstrated a null association in at least 80% of included primary studies. |
| “0”  (Less consistent null association) | The variable shows a null association in at least 60% of reviews. It also means that either a single review included the variable and demonstrated a null association in at least 60% of included primary studies. |
| “?”  (Unclear or contradictory) | If there is no agreement in the direction of association for at least 60% of reviews, or included primary studies, the direction of association is graded as unclear or contradictory. |
| “-”  (Less consistent negative association) | A variable shows a significant negative association or reports effect sizes (RR) lower and equals to 0.66 in at least 80% of reviews. If only a single review is available, the variable shows a significant negative association or effect sizes less than 0.66 in at least 80% of the included primary studies. |
| --  (Consistent negative association) | A variable shows a significant negative association or reports effect sizes (RR) lower than and equals to 0.66 in at least 60% of reviews. If only a single review is available, the variable shows a significant negative association or effect sizes less than and equals to 0.66 in at least 60% of the included primary studies. |

# **Supplementary Table S4: Strength of evidence grading criteria**

| **Strength of evidence**^2,3^ | **Grading criteria** |
| --- | --- |
| Convincing evidence  (Ce) | Evidence strong enough to support a causal or protective relationship, justifying recommendations to reduce neonatal mortality. Unlikely to change with future research.  **All of the following required:**   - Evidence from ≥2 independent cohort studies **and** at least one other study type. - No substantial unexplained heterogeneity (I² < 50%). - Consistent association: ≥80% of studies in same direction of effect. - High methodological quality to confidently exclude that the observed association results from random or systematic error, including confounding, measurement error, and selection bias. - Plausible biological gradient (dose–response), if applicable, even if not linear or uniform across exposure levels. - Supporting experimental evidence, where available |
| Probable evidence  (Pe) | Evidence sufficiently strong to support a probable causal or protective relationship, generally justifying recommendations to reduce neonatal mortality. However, limitations in the evidence base prevent a more definitive conclusion. Limitations of the evidence may include substantial heterogeneity (I² ≥ 50%), insufficient study duration, a limited number of studies or inadequate sample sizes, and incomplete follow-up.  All of the following are generally required:   - Evidence from at least two independent cohort studies or five case-control studies. - Consistent associations with ≥60% agreement in the direction of effect between the variable and neonatal mortality. - Studies of good methodological quality, minimising the likelihood that observed associations result from random or systematic error, including confounding, measurement error, or selection bias. |
| Limited suggestive evidence (Ls) | Evidence is too limited to permit a probable or convincing causal judgment but is suggestive of a direction of effect. Limitations may include a small number of studies or methodological flaws, with evidence primarily based on cross-sectional designs. Such evidence rarely justifies recommendations to reduce neonatal mortality. Insufficient longitudinal observational or experimental studies are available, or results are inconsistent. Additional well-designed studies are needed to support these tentative associations. |
| Limited, no conclusive evidence (Lnc) | Evidence is currently too limited to draw firm conclusions. Limitations may arise from a small number of studies, inconsistent directions of effect, methodological flaws, or a combination of these factors. A classification of limited–no conclusive evidence does not imply the absence of an association; with further high-quality research, the factor may be shown to increase or decrease the risk of neonatal mortality. Present evidence is based on a few suggestive studies that are insufficient to establish a clear association, with no available longitudinal observational or experimental data. Additional rigorous, well-designed studies are required to clarify these potential associations. |

#

# **Supplementary Table S5: Formula used for converting odds ratio (OR) to relative risk (RR)**

|  | **Formula and descriptions** |
| --- | --- |
| Formula for converting OR to RR | RR=$\frac{OR}{1+p\left( OR-1 \right)}$  where p is the prevalence among the control population.^4^ |

# **Supplementary Table S6: Excluded reviews and reasons for exclusion after full-text review**

| **Reviews** | **Reasons** |
| --- | --- |
| Ahmed 2017^5^ | Wrong estimate: the study reviewed the magnitude of neonatal mortality |
| Ahrens 2018^6^ | Reported neonatal mortality based on a single study |
| Asferie 2025^7^ | Wrong outcome; did not report neonatal mortality |
| Bell 2014^8^ | Wrong publication: this is a commentary. |
| Bitew 2020^9^ | Wrong population; included neonates from neonatal intensive care unit |
| Brinchmann 2023^10^ | Wrong outcome; did not report neonatal mortality |
| Di 2021^11^ | Wrong outcome; did not report neonatal mortality |
| Daemi 2019^12^ | Wrong estimate, the study reported correlation |
| Go 2011^13^ | Wrong publication (commentary) |
| Gissler 2009^14^ | The finding for neonatal mortality was not clear. It was mixed with the finding of stillbirth and infant mortality. |
| Hessami 2022^15^ | Wrong population, conducted among special population |
| Hardee 2021^16^ | Wrong population |
| Ho 2021^17^ | Wrong outcome, reported cause specific mortality not all cause mortality |
| Hodgkin 2010^18^ | Wrong population, conducted among special population |
| Jans 2010^19^ | Wrong population, conducted among special population |
| Jia 2024^20^ | Wrong outcome; did not report neonatal mortality |
| Khan 1999^21^ | Wrong publication, this is a commentary. |
| Kim 2013^22^ | Wrong outcome; did not report neonatal mortality |
| Kontovazainitis 2023^23^ | Wrong estimate |
| Kozuki 2013^24^ | Wrong exposure (assessed combined exposure) |
| Lo 2024^25^ | Wrong outcome; did not report neonatal mortality |
| Lalani 2018^26^ | Wrong population, included special population |
| Marchi 2015^27^ | Wrong outcome; did not report neonatal mortality |
| Martinez-Hortelano 2024^28^ | Wrong outcome; did not report neonatal mortality |
| Murphy 2013^29^ | Replaced by updated review |
| Mersha 2019^30^ | Wrong outcome; did not report neonatal mortality |
| Ni 2023^31^ | Wrong outcome; did not report neonatal mortality |
| Pastor-Moreno 2020^32^ | Wrong outcome; did not report neonatal mortality |
| Pinheiro 2019^33^ | Wrong outcome; did not report neonatal mortality |
| Pratiwi 2020 ^34^ | Wrong exposure, examined cause of neonatal mortality |
| Ramaiya 2014^35^ | Wrong outcome; did not report neonatal mortality |
| Saccone 2022^36^ | Incorrect outcome; did not report neonatal mortality |
| Saputri 2024^37^ | Wrong exposure definition and mixed results |
| Shen 2024^38^ | Wrong population, conducted among special population |
| Shi 2024^39^ | Full text articles not available |
| Tran 2015^40^ | Wrong comparison |
| Vallely 2021^41^ | Wrong outcome; did not report neonatal mortality |
| Veenendaal 2011^42^ | Wrong outcome; did not report neonatal mortality |
| Walther 2021^43^ | Wrong population, conducted among special population |
| Wang 2021^44^ | Wrong outcome; did not report neonatal mortality |
| Wang 2022^45^ | Wrong outcome; did not report neonatal mortality |
| Wendt 2012^46^ | Wrong outcome; did not report neonatal mortality |
| Yan 2012^47^ | Full text articles not available |
| Yang 2023^48^ | Wrong population, conducted among special population |
| Ye 2022^49^ | Incorrect outcome; did not report neonatal mortality |
| Yitayih 2023^50^ | Incorrect outcome; did not report neonatal mortality |
| Zulu 2024^51^ | Incorrect outcome; did not report neonatal mortality |

# **Supplementary Table S7: Characteristics of systematic reviews and meta-analyses**

| **First author, (No. of authors, countries)** | **Risk factors** | **No. of databases, grey literatures** | **Search date range and language applied** | **Publication year range of included studies** | **No. of primary studies, study design, coverage** | **No. of ineligible studies included in the UR; study design** | **Total no. of participants; no. of participants in eligible studies** | **Risk of bias assessment tool** | **Reporting guideline** | **Evidence of pre-specified protocol** | **Quality of the review** |
| --- | --- | --- | --- | --- | --- | --- | --- | --- | --- | --- | --- |
| Afraie 2023^52^ (5; all Iran) | HBV infection | DB = 7  GL=Yes | January 1990 – February 2023. NA | 2003–2020 | 35: all cohort. Global | 6; all cohort | 34,140,943 participants; 182,421 participants | NOS | PRISMA | No | Critically low |
| Ashworth 2014^53^ (3; all UK) | Waste incineration | DB =1  GL= No | NA.  English | 1988–2009 | 14; 9 geographic study, 3 cohort, 2 case-control. Global | 2; 1 retrospective cohort, 1 geographic study | 743,356* participants; 3,134 deaths | STROBE  checklist | NA | No | Critically low |
| Aune 2014^54^ (4; 2 Norway, 1 UK, 1 USA) | Maternal body mass index | DB = 2  GL= No | Inception – January 23, 2014. NA | 1992–2014 | 38: all cohort. Global | 12: all cohort | NA; 3,321,555 participants | MNOS | NA | No | Low |
| Aynalem 2021^55^ (6: all Ethiopia) | Residence, Preterm birth | DB = 6  GL=Yes | NA. English | 2014–2019 | 12; 9 cohort, 3 cross-sectional. Ethiopia | 12; 9 cohort, 3 cross-sectional | 12,397 participants | MNOS | PRISMA | No | Critically low |
| Balaj 2021^56^ (19; 10 USA, 9 Norway) | Maternal education, paternal education | DB =5  GL=Yes | Inception – January 2021. No language restriction | 1982–2020 | 300; unclear.  Global | 300†; NA | 3,112,474* participants; NA | NA | PRISMA | Yes | Moderate |
| Behboudi-Gandevani 2022^57^ (7; 3 Norway, 3 Iran, 1 Austria) | Immigration | DB = 3  GL = No | Inception – September 2020. English | 1999–2020 | 45; NA. Global | 14; NA | 48,533,304 women; 22,204,603 women | NOS | PRISMA | No | Critically low |
| Belachew 2020^58^ (3 all Ethiopia | Complete ANC uptake | DB = 8  GL = Yes | Inception – 01 October 2018. English | 1912–2018 | 11; 4 cohort, 7 cross-sectional. Ethiopia | 11; 4 cohort, 7 cross-sectional. | 34,088 participants | NOS | PRISMA | No | Critically low |
| Boafor 2015^59^ (13; 6 Ghana, 6 USA, 1 Nigeria) | Sickle-cell disease | DB = 1  GL = No | January 2000 – October 2014. English | 2001–2013 | 16; 1 RCT, 2 cohort, 13 case-control.  Low- and high-income countries | 6; 2 cohort, 4 case-control | NA; 2,659 participants | NA | NA | No | Low |
| Brocklehurst 1998^60^ (2; all UK) | Maternal HIV infection | DB = 3  GL = No | 1983 – December 1996. NA | 1988–1996 | 31: all cohort. Global | 3; all cohort | 46,878 women; 1,517 women | A predefined set of criteria | NA | No | Critically low |
| Chaka 2019 ^61^(5; all Iran) | Health facility delivery | DB >5  GL = Yes | Inception – February 2019. No language restriction | NA | 19; 5 cohort, 8 case-control, 6 cross-sectional. Global | 19; 5 cohort, 8 case-control, 6 cross-sectional | 1,045,562 live births | MNOS | PRISMA | No | Moderate |
| Chmielewska 2021^62^ (15; 9 UK, 6 Turkey) | COVID 19 pandemic | DB = 2  GL = No | January 1st, 2020 –January 8th, 2021. No language restriction | 2020–2021 | 40; all retrospective cohort. Global | 3; NA | 3,461,877 pregnancies; 93,111 pregnancies | NOS | PRISMA | Yes | Low |
| Debes 2013^63^ (5; 4 USA, 1 Australia) | Early breastfeeding initiation | DB = 6  GL = Yes | 1963 – 2011. No language restriction | NA | 18; 1 randomized trial, 11 cohort, 4 case-control, 2 cross-sectional.  Global | 3; all cohort | NA; 44,249 participants | Child Health Epidemiology Reference Group guidelines | NA | No | Critically low |
| Deng 2024^64^ (16; all China) | History of stillbirth | DB = 5  GL = No | Inception – July 2023.  English, Chinese | 2001–2022 | 19; all cohort. Global | 7; all cohort | 4,855,153 participants; 362,300 participants | MNOS | PRISMA | Yes | Moderate |
| Desta 2021^65^ (8; all Ethiopia) | Pre-term birth | DB = 7  GL = Yes | Inception –July 27, 2020. English | 2008–2020 | 33; 7 cohort, 8 case-control, 16 cross-sectional. Ethiopia | 9; 6 cohort, 3 cross-sectional | 20,109 live births; 10,149 live births | NOS | PRISMA | No | Critically low |
| Downes 2017^66^ (3; all USA) | Placental abruption | DB = 5  GL = No | January 1^st^, 2005 – December 31^st^, 2016.  English | 2005–2016 | 123; NA  Global | 3; all cohort | NA | NA | PRISMA | No | Critically low |
| Glinianaia 2004^67^ (5; all UK) | Ambient air pollution | DB = 14  GL = Yes | January 1, 1966 – December 31, 2003.  English | 1972–2003 | 15; 2; cohort, 1 case-control, 10 ecologic or time series, 2 cross-sectional.  Global | 6; 1 case-control, 1 cross-sectional, 4 ecologic | 13,000,000* live births; NA | NA | U.K. National Health Service Centre for Reviews and Dissemination | No | Critically low |
| Graeve 2022^68^ (8; 4 Germany, 4 USA) | Prenatal exposure to opioids | DB = 4  GL = No | Inception – March 1^st^, 2021.  English | 1974–2020 | 80; 75 cohort, 3 case-control, 2 cross-sectional.  Global | 14; 12 cohort, 2 case-control | 23,670,404 participants; 522,073 participants | NOS | PRISMA, MOOSE | Yes | Moderate |
| Hall 2017^69^ (4; all UK) | Unintended pregnancy | DB = 4  GL = No | 1975 – March 2015.  English, French, Spanish | 1980–2015 | 84; NA.  Global | 5; NA | 593,735* participants; 90,091 participants | NA | MOOSE | No | Critically low |
| Hassen 2024^70^ (7; 1 Ethiopia, 5 Australia, 1 Bangladesh ) | Short interpregnancy interval | DB = 5  GL = No | September 2000 – May 2023. English | 2001–2023 | 41; 6 case-control, 12 cohort, 23 cross-sectional.  Asia-Pacific  region^70,71^ | 6; all cross-sectional | 1,770,339 participants; 105,291 participants | JBI | PRISMA | Yes | Low |
| He 2020^72^ (4; all China) | Maternal chlamydia trachomatis | DB = 4  GL = No | Inception – December 9, 2019.  English | 1980–2019 | 50; 21 cohort, 29 case-control. Global | 3; all cohort | 502,141 participants; NA | MNOS | NA | No | Low |
| Helmyati 2022^73^ (7; all Indonesia) | Complete ANC uptake | DB = 5  GL = Yes | January 1990 – May 2021. Bahasa Indonesia, English | 2010–2022 | 24; 8 cohort, 16 case-control.  Indonesia | 4; 1 cohort, 3 case-control | 57,671* participants; 12,032 participants | NOS | PRISMA | No | Low |
| Hulse 1998^74^ (4; all Australia) | Maternal opiate use | DB = 1  GL = No | 1966 – June 1996.  English | 1971–1987 | 7; NA.  Global | 6; 4 cohort, 2 NA | 120335 participants; 120,227 participants | NA | NA | No | Critically low |
| Huo 2021^75^ (7; 6 China, 1 Germany) | Maternal body mass index | DB = 3  GL = No | Inception – November 26, 2020.  No language restriction. | 2001–2019 | 22; all cohort.  Global | 13; all cohort | 13,532,293 participants; 3,848,782 participants | NOS | MOOSE | No | Low |
| Islam 2022^71^ (7; 3 Bangladesh, 1 Malaysia, 1 Australia) | Short interpregnancy interval | DB = 8  GL = No | January 2000 – January 2022.  English | 2010–2021 | 56; 3 cohort, 3 case-control, 45 cross-sectional.  LMICs | 17; 1 cohort. 16 cross-sectional | 869,615* participants; 279,345* participants | NOS | PRISMA, STROBE | No | Low |
| Jahan 2007^76^ (1; Saudi Arabia | Poverty | DB = 4  GL = No | Inception – February 2007. English | 1992–2006 | 9; 2 case-control, 7 cross-sectional. Eastern Mediterranean region | 3; 1 case-control, 2 cross-sectional | NA | NA | NA | No | Critically low |
| Jansen 2023^77^ (8; all Netherland) | Hyperemesis gravidarum | DB = 2  GL = No | Inception – February 9th, 2022. No language restriction | 1957–2021 | 61; 48 cohort, 13 case-control.  Global | 5; all cohort | 20,532,671 participants; 3,973,154 participants | NOS | PRISMA | Yes | Critically low |
| Jung 2019^78^ (7; 6 Japan, 1 Bangladesh ) | Anaemia during pregnancy | DB = 4  GL = No | Inception – November 21, 2018. No language restriction | 1981–2018 | 117; 88 cohort, 29 case−control. Global | 5; all cohort | 4,127,430 pregnant women; 214,747 pregnant women | NOS | PRISMA | Yes | Moderate |
| Karami^79^ 2024^79^ (3; all Iran) | Socio-demographic and economic factors, maternal reproductive health-related factors, child-related factors | DB = 5  GL = Yes | Inception - January 2022. English, Farsi | 2001–2022 | 32; 8 case-control, 21 cross-sectional, 2 time-series, 1 ecological. Iran | 32†: NA | NA | STROBE | PRISMA | No | Low |
| Keag 2018^80^ (3; 2 UK, 1 Australia) | Caesarean section delivery | DB = 4  GL = No | Inception – May 25, 2017. No language restriction | 2001–2016 | 80; 1 RCT; 79 cohorts. HICs | 5; all cohort | 29,928,274 participants; 10,275,127 participants | Scottish Intercollegiate Guideline Network | MOOSE | Yes | Low |
| Khan 2015^81^ (4; 2 Switzerland, 1UK, 1 Sweden) | Timing of breastfeeding initiation | DB = 2  GL = No | Inception – February 2012. NA | 1982–2011 | 11; 9 cohort, 2 case−control. Global | 11; 9 cohort, 2 case−control. Global | 70,976 participants | GRADE | PRISMA | No | Critically low |
| Kozuki 2013^82^ (9; 6 USA, 2 Brazil, 1 Zimbabwe) | Short interpregnancy interval | DB = 2  GL = Yes | NA | 1983–2005 | 5; all cohort. LMICs | 5; all cohort. | 32,670 singleton live births | NA | NA | No | Critically low |
| Lean 2017^83^ (4; all UK) | Advanced maternal age | DB = 5  GL = No | Since 2000.  English | 2000–2015 | 72; 59 cohort, 13 case-control.  Global | 27; 15 cohort, 12 case-control | 51,752,249 births; 13,245,799 births | Question developed by Sanderson et al., 2017 | PRISMA, MOOSE | No | Critically low |
| Li 2021^84^ (6; all China) | Hypertensive disorder of pregnancy | DB = 7  GL = No | Inception – May 2019. English, Chinese. | 1983–2019 | 152; All cohort.  Global | 33: all cohort | 37,801,284 participants; 4,027,092 participants | NOS | PRISMA, MOOSE | No | Low |
| Maraka 2016^85^ (10; all USA) | Sub-clinical hypothyroidism | DB = 5  GL = No | Inception –January 2015. No language restriction | 2005–2014 | 18; All cohort.  Global | 6; all cohort | 3995 cases; NA | NOS | PRISMA | No | Critically low |
| Mengistu 2020^86^ (5; all Australia) | Severe maternal morbidity, hypertensive disorder, haemorrhagic disorder, hepatic disorder | DB = 4  GL = No | NA. English | 1994–2018 | 35; 26 cohort, 8 case-control, 1 cross-sectional.  HICs | 13; 9 cohort, 4 case-control | 38,909,426 women; 6,699,953 women | NOS | PRISMA | Yes | Low |
| Muglu 2019^87^ (10; 8 UK, 1 Spain, 1 Malaysia) | Pre-term birth | DB = 3  GL = Yes | January 1990 – October 2018.  No language restriction | 1990–2015 | 13: all cohort.  HICs | 5; all cohort | 15,124,027 pregnancies; 7,194,423 births | NA | PRISMA | Yes | High |
| Nguyen 2019^88^ (7; 5 Australia, 2 USA) | Maternal death | DB = 3  GL = Yes | January 1980 – March 2017. English | 2003–2015 | 12; NA. LMICs | 4; all cohort | 702,112 participants; 452,699 participants | NA | PRISMA | No | Low |
| Pineles 2015^89^ (4; all USA) | Maternal smoking during pregnancy | DB = 1  GL = No | 1956 –August 31, 2011.  No language restriction | 1959–2011 | 142; 110 cohort, 25 case-control, 7 cross-sectional.  Global | 45; NA | NA | NA | PRISMA, MOOSE | No | Critically low |
| Quansah 2015^90^ (11; 2 Finland, 6 Ghana, 2 Canada, 1 China) | Arsenic exposure | DB = 3  GL = No | 1946 – July 2013. NA | 1989–2013 | 23; NA.  Global | 5; 3 cohort, 2 cross-sectional | 67,426* participants; 39,759* participants | NOS | PRISMA | No | Low |
| Rahman 2016^91^ (9; all Japan) | Maternal anaemia | DB = 3  GL = No | Inception – May 2015.  No language restriction | 1994–2014 | 29: All cohort.  LMICs | 2; all cohort | 720,000 participants; 1242 participants | NOS | PRISMA, MOOSE | No | Low |
| Rahman 2020^92^ (3; all Bangladesh) | Maternal anaemia | DB = 3  GL= No | Inception –February 20, 2020.  No language restriction | 1992–2018 | 35; 20 cohort, 8 case control, 7 cross-sectional. South Asia | 2; all cohort | 209,796 participants; 160,585 participants | NOS | PRISMA | No | Critically low |
| Rathore 2022^93^ (13; 2 India, 1 Saint Vincent and the Grenadines, 1 China, 2 Pakistan, 2 Barbados, 3 Colombia, 1 Ghana, 1 Saudi Arabia) | Dengue virus infection during pregnancy | DB = 3  GL = Yes | Inception –December 10, 2021.  No language restriction | 2000–2021 | 36; 34 cohort, 1 case-control, 1 cross-sectional.  Global | 4; all cohort | NA; 4161 participants | NOS | PRISMA | No | Critically low |
| Robjin 2024^94^ (12; 8 Australia, 3 USA, 1 China) | Maternal asthma | DB = 4  GL = No | March 2012 – September  2023. English | 1990-2023 | 40; all cohort. Global | 8; all cohort | 17,054,798 participants; 241,715 participants | NOS | PRISMA, MOOSE | Yes | High |
| Shah 2011^95^ (4; all Canada) | Indigenous status (Aboriginal/non-aboriginal) | DB = 3  GL = No | Inception – September 2009.  English | 1973–2010 | 38; All cohort. Australia, Canada, USA | 12; all cohort | 32,799,670 participants | Predefined checklist | MOOSE | No | Low |
| Shen 2023^96^ (5; All China) | Hepatitis C infection | DB = 5  GL = Yes | January 1, 1950 – October 15, 2022. No language restriction | 2000–2022 | 14; 12 cohort, 2 case-control. Global | 2; all cohort | 5,655,361 pregnant women; 1,448,480 pregnant women; | NOS | PRISMA | Yes | Moderate |
| Smith 2017^97^ (6; 2 USA, 1 UK, 2 India, 1 Australia) | Breastfeeding initiation | DB = 8  GL = No | Inception – December 2015. NA | 1990–2015 | 11; 8 cohort, 2 case-control, 1 cross-sectional.  Global | 6; all cohort | >159,603* participants | Criteria developed in accordance with the World Health Organization Child Health Epidemiology Reference Group | MOOSE | Yes | Low |
| Tang^98^ (9; 2 China, 7 Canada) | Interpregnancy Weight gain | DB = 4  GL = No | Inception –July 10, 2024. English | 2006–2022 | 13 NA. Global | 4; all cohort | 3,569,695 women; | ROBINS-I | PRISMA | Yes | High |
| Tekelab 2019^99^ (4; all Australia) | Antenatal care | DB = 6  GL = Yes | Inception – April 13, 2019. English | 2009–2019 | 12; 5 cohort, 2 case-control, 5 cross-sectional.  SSA | 12; 5 cohort, 2 case-control, 5 cross-sectional. | 79,990 live births | RoBANS | PRISMA | Yes | Low |
| Thomson 2021^100^ (13; all UK) | Maternal occupational status | DB = 9  GL = Yes | 1999 –August 2019. No language restriction | 1997–2019 | 35; 25 cohort, 10 case-control  UK, Republic of Ireland | 3; all cohort | 17,473,374* participants; 9,218,373 participants | GRADE | MOOSE | Yes | Low |
| Tiruye 2022^101^ (5; all Ethiopia) | Antenatal care | DB = 6  GL = Yes | January 1st, 1990 – February 12th, 2021.  English | 2011–2020 | 27; 12 cohort, 5 case-control, 10 cross-sectional.  East Africa | 27; 12 cohort, 5 case-control, 10 cross-sectional. | 85,530 participants | RoBANS | PRISMA | No | Critically low |
| Tiruye 2023^102^ (2; all Ethiopia) | Antenatal care | DB = 6  GL = Yes | January 1990 – June 2020.  English | 1997–2020 | 28; 13 cohort, 7 case-control, 8 cross-sectional.  Ethiopia | 28; 13 cohort, 7 case-control, 8 cross-sectional. | 61,730 participants | RoBANS | PRISMA | No | Critically low |
| Tolossa 2020^103^ (6; all Ethiopia) | Antenatal care | DB = 6  GL = Yes | NA. English | 1997–2019 | 14; 6 cohort, 5 case-control, 3 cross-sectional.  Ethiopia | 14; 6 cohort, 5 case-control, 3 cross-sectional. | 23,932 participants | JBI | PRISMA | No | Critically low |
| Tura 2013^104^ (3; all Ethiopia) | Health facility delivery | DB = 3  GL = Yes | 1980 –October 2012. English | 1988–2012 | 19; 1 community trial, 5 cohort, 4 case-control, 9 cross-sectional.  Global | 19; 1 community trial, 5 cohort, 4 case-control, 9 cross-sectional | 1,606,805 live births | NA | PRISMA | No | Critically low |
| Veloso 2018^105^ (7; all Brazil) | Maternal education, maternal marital status, maternal age, neonate sex, history of still birth, multiple pregnancy, complication during pregnancy, birth weight congenital malformation, low APGAR at 5th minute, gestational age, caesarean section delivery | DB = 6  GL = Yes | 2000–2018.  English, Portuguese | NA | 10; 4 cohort, 6 case-control.  Brazil | 10; 4 cohort, 6 case-control | 213,250 participants | NOS | NA | Yes | Moderate |
| Wedi 2016^106^ (6; all UK) | Untreated maternal HIV infection | DB = 9  GL = No | January 1^st^, 1980 – December 7^th^, 2014.  No language restriction | 1989–2014 | 35; 32 cohort, 3 case-control.  Global | 3; all cohort | 53,623 women; 2,297 women | NOS | PRISMA | Yes | High |
| Weightman 2012^107^ (6: all UK) | Area deprivation, social class | DB = 23  GL = Yes | 1994 – May 2011. No language restriction | 1994-2010 | 36; 30 cohort, 6 case-control.  UK | 3; all cohort | NA; 21,239,008 live births | NOS | NA | No | Low |
| Wondemagegn 2018^108^ (4; all Ethiopia) | Focused antenatal care | DB = 7  GL = Yes | NA. English | 2009-2017 | 18; 1 community trial, 4 cohort, 4 case-control, 9 cross-sectional. Global | 18; 1 community trial, 4 cohort, 4 case-control, 9 cross-sectional. | 156,512 births | NA | PRISMA | No | Critically low |
| Wulandari 2021^109^ (3; all Indonesia) | Maternal obesity | DB = 5  GL = Yes | 2010 – 2021. English | 2011–2020 | 19; 15 cohort, 4 cross-sectional  Global | 8; 4 cohort, 4 cross-sectional | 6145491 pregnant women; 464,989 pregnant women | Critical Appraisal Checklist cohort and cross-sectional studies | PRISMA | No | Critically low |
| Xie 2021^110^ (3; all China) | Maternal psoriasis | DB = 3  GL = No | Inception – 3^rd^ August 2020. No language restriction | 2007–2020 | 16; 14 cohort, 2 case-control.  Global | 3; 2 cohort, 1 case-control | 8,044,996 pregnancies; 1,756,215 pregnancies | NOS | PRISMA | No | Low |
| Yang 2022^111^ (6; all Canada) | COVID 19 pandemic | DB = 2  GL = No | Inception–November 20, 2021. English | 2019-2021 | 54; NA.  Global | 3; NA | 30,890,821 pregnancies; 93111 pregnancies | NOS | PRISMA | Yes | Low |
| Yarandi 2021^112^ (5; 3 Norway, 2 Iran) | Mild gestational diabetes mellitus | DB = 3  GL = No | Inception – May 2020. English | 2000–2019 | 17; 15 cohort, 2 interventional. Global | 2; NA | 64,680 pregnant women; 1889 pregnant women | NOS | PRISMA | No | Critically low |
| Young 2023^113^ (7; 6 USA, 1 Germany) | Maternal haemoglobin | DB = 2  GL = No | January 1990–April 2021. No language restriction | 1990-2022 | 148; 93 cohort, 36 case-control, 19 cross-sectional. Global | 10; 8 cohort, 1 case-control, 1 cross-sectional | 13,839,327 women; 1,197,735 women | NA | NA | No | Critically low |
| Younger 2022^114^ (5; all USA) | household air pollution from unclean cooking fuel | DB = 4  GL = No | May 1, 2013–June 12, 2021. English, Spanish | 2013-2021 | 23; 3 randomised control trial, 5 cohort, 1 case-control, 14 cross-sectional. LMICs | 6; 1 RCT, 1 cohort, 4 cross-sectional | 362668 participants; 191,645 participants | Office of Health Assessment and Translation tool | PRISMA | Yes | High |
| Yu 2017^115^ (15; all China) | Pre-gestational diabetes mellitus | DB = 5  GL = No | January 1990–February 2017, no language restriction | 1994-2017 | 100; 67 cohort, 30 case-control, 3 cross-sectional. Global | 19; 16 cohort, 3 case-control | >40,000,000 participants; 7,634,750 participants | NOS | MOOSE | No | Low |
| *The number of participants was incomplete. †The review did not provide the number of studies included for neonatal mortality. DB = database. GL = grey literature. GRADE = Grading of Recommendations, Assessment, Development and Evaluation. HICs = High income countries. JBI = Joanna Briggs Institute Critical Appraisal tool. LMICs = low- and middle-income countries. MOOSE = The Meta-analysis of Observational studies in Epidemiology. NA = not available. NOS = Newcastle Ottawa Scale. PRISMA = The Preferred Reporting Items for Systematic Review and Meta-analysis. RoBANS = Risk of Bias Assessment tool for Non-randomized Studies. STROBE = Strengthening the Reporting of Observational Studies in Epidemiology. | | | | | | | | | | | |

# **Supplementary Table S8: Summary results from systematic reviews and meta-analyses**

| **First author** | **Summary results** |
| --- | --- |
| Afraie 2023^52^ | Hepatitis B virus infection:  Six studies including 182,421 participants, RR 0.83 [95% CI 0.67, 1.03], I^2^ =0.0%, p-value = 0.95. |
| Ashworth 2014^53^ | Waste incineration:  Two studies (one retrospective cohort and one geographic study) including 2,849 births reported associations including null values in their confidence interval. One study OR 1.03 [95% CI 0.93–1.13], one study OR = 0.91 [95% 0.25–2.32] |
| Aune 2014^54^ | Maternal Body Mass Index:  Twelve cohort studies were included in the analysis of maternal BMI and neonatal death and included 11,294 deaths among 3,321,555 participants. The summary RR per 5 BMI units was 1.15 [95% CI 1.07–1.23]; I^2^ = 78.5%; P<.001. There was no evidence of publication bias with the Egger test (P = 0.18). |
| Aynalem 2021^55^ | Gestational age:  Seven studies, OR 1.32 [95% CI 1.07–1.58]; I^2^ = 38%, no evidence of publication bias, Begg’s test (p = 0.37) and Egger’s test (p = 0.7).  Residence:  Six studies, OR 1.93 [95% CI 1.13–2.73]; I^2^ = 92%; no evidence of publication bias, Begg’s test (p = 0.99) and Egger’s test (p = 0.663). |
| Balaj 2021^56^ | Maternal education:  Teritiary education vs no education, RR 0.83 [95% CI 0.82–0.86].  Paternal educational status:  Teritiary education vs no education, RR 0.88 [95% CI 0.87–0.91]. |
| Behboudi-Gandevani 2022^57^ | Immigrant mother:  Fourteen studies involving 5,216,619 immigrant women and 16,991,984 native-origin women, OR 1.09 [95% CI 1.00–1.19); I^2^ = 93.6%; no publication bias, stable with sensitivity analysis. |
| Belachew 2020^58^ | Complete antenatal care:  Having 4 or more ANC visit vs. less than 4 visit), 3 studies, OR 0.57 [95% CI 0.42–0.77); I^2^ = 84.1%; p = 0.002 |
| Boafor 2015^59^ | Sickle-cell disease:  Six studies involving 2659 participants, OR 2.71 [95% CI 1.41–5.22]; no heterogeneity. This risk is higher among babies of women with SCD in high income, OR 2.43 [95% CI 1.20–4.96], but not in low-income countries, OR 4.50 [95% CI 0.93–26.87]. |
| Brocklehurst 1998^60^ | Maternal HIV infection:  Three studies including 766 HIV-infected and 751 HIV-uninfected women, OR 1.10 [95% CI 0.63–1.93]; heterogeneity and publication bias not reported. |
| Chaka 2019 ^61^ | Health facility delivery:  Nineteen studies, OR 0.48 [95% CI 0.38–0.58]; I^2^ = 84.5%; Egger's test p = 0.006, significant publication bias. |
| Chmielewska 2021^62^ | COVID 19 pandemic:  Three studies including 93,111 participants , OR 1.01 [95% CI 0.38–2.67], I^2^ = 85%; P < 0.001 |
| Debes 2013^63^ | Early initiation of breastfeeding:  Three prospective cohort studies involving 44,249 participants, RR = 0.56 [95% CI 0.40–0.79] |
| Deng 2024^64^ | History of stillbirth:  Seven studies including 362,300 participants, OR 4.24 [95% CI 2.65–6.79]; I^2^ = 47%. Publication bias not assessed for the neonatal mortality. |
| Desta 2021^65^ | Preterm birth: Nine (6 cohort and 3 cross-sectional studies), including 10149 participants, OR 3.16 [95% CI 1.57–6.34]; I^2^ = 95.7%; p = 0.000, no publication bias based on the Eggers test and Funnel plot. |
| Downes 2017^66^ | Placental abruption:  Two cohort studies (one study Singletons: OR 11.1 [95% CI 10.0–12.3] , Twin: OR 4.2 [95% CI 3.7–4.8], Triplets: OR 2.0 [95% CI 1.1–3.5]; 1 study singletons: OR 0.9 [95% CI 0.5–1.7]. |
| Glinianaia 2004^67^ | Air pollution:  Seven studies, majority of studies reported an increased risk of neonatal mortality with increased ambient air pollution.  Per 10-μg/m3 increase in PM10, OR 1.13 [95% CI 1.09–1.18].  Per 10-μg/m3 increase in TSP, OR 1.00 [95% CI 0.96–1.06].  > 84.7 (top quintile) vs. < 53.6 μg/m3 TSP (bottom quintile): Total neonatal, OR 1.18 [95% CI 1.00–1.39]. |
| Graeve 2022^68^ | Opioid exposure during prenatal period:  Fourteen studies including 522,073 participants, RR 4.05 [95% CI 2.12–7.72]; I^2^ =73%. |
| Hall 2017^69^ | Unintended pregnancy:  Five studies, one study OR 1.83 [95% CI 1.01–3.34], one study OR mistimed vs wanted 1.82 [95% CI 1.16–2.84], OR unwanted vs wanted 2.22 [95% CI 1.17–4.24], one study OR 2.09 (p < 0.001), one study RR: 2.4 [95% CI 1.5–4.0]. |
| Hassen 2024^70^ | Short interpregnancy interval:  Six cross-sectional studies, OR: 1.78 [95% CI 1.25–2.55]; I^2^ =95.2%, egger test p-value = 0.00 |
| He 2020^72^ | Chlamydia trachomatis:  There were three cohort OR 0.99 [95% CI 0.49–2.02] and one case–control studies OR 2.00 [95% CI 0.46–0.60]. Two types of studies were not merged due to different directions. The case–control study indicated no significant association between C. trachomatis infection and neonatal death OR 2.00 [95% CI 0.46–8.60]; p = 0.352, so did cohort studies OR 0.99 [95% CI 0.49–2.02); p = 0.98 |
| Helmyati 2022^73^ | Complete antenatal care:  Four studies with low risk of bias, one cohort and 3 case-control including 12,032 live births, RR: 2.59 [95% CI 1.01–6.66]; I^2^=93% p<0.00001; no publication bias. |
| Hulse 1998^74^ | Maternal opiate use:  Any opiate use: 6 studies including 120, 227 participants, RR 2.73 [95% CI 1.41–5.28]. Heroin use, RR 1.47 [95% CI 0.88–2.33]. Methadone use 1.75 [95% CI 0.60–4.59]. Heroin and methadone use 6.37 [95% CI 2.57–14.68). Any methadone use (with or without heroin use), RR 3.00 [95% CI 1.50–5.88]. |
| Huo 2021^75^ | Maternal body mass index:  Compared to normal weight women, Neonates of overweight and obese women had higher risk of neonatal mortality, but however children of Underweight women had no significant risk for neonatal mortality. Maternal underweight: 12 studies involving 3,848,782 participants, RR 1.08 [95% CI 0.93–1.26]; I^2^ = 16%; P=0.288. Maternal overweight: 12 studies involving 3,848,782 participants, RR 1.23 [95% CI 1.08–1.39], I^2^ = 62.4%; P=0.001. Maternal obesity: 12 studies involving 3,848,782 participants, RR 1.55 [95% CI 1.28–1.67], I^2^ = 75.6%, p=0.000. Egger’s test (p = 0.099). In subgroup analysis on infant mortality (obesity vs. normal), the studies reporting infant death cases > 500 and those published after 2000 might partially contribute to the heterogeneity, while meta-regression did not identify any potential source of heterogeneity. In the subgroup analysis on neonatal mortality (overweight vs. normal), studies from Europe and those published before 2000 might partially contribute to the heterogeneity. Leave-one-out sensitivity analyses No significant change after studies were omitted one by one, showing consistent with overall findings. |
| Islam 2022^71^ | Short interpregnancy interval (<24 months)  Seventeen studies (>279,345 participants, one study not reported)) OR 1.85 [95% CI 1.68, 2.04], I^2^ = 72.6%, Egger's test (p: <0.01), 3 studies were missing, Trim and Fill estimates: OR 1.76 [95% CI 1.60–1.95]. |
| Jahan 2008^76^ | Poverty:  Three studies reported neonatal deaths. For both SES and maternal illiteracy, OR 1.48 [95% CI 1.17–1.87], maternal illiteracy, OR 1.66 [95% CI 1.11–2.48] and for low SES OR 1.26 [1.12, 1.42). |
| Jansen 2023^77^ | Hyperemesis gravidarum :  Five studies (4 cohort, 1 case control) involving 3,973,154 participants shoed insufficient evidence on the association between hyperemesis gravidarum and neonatal death, OR 1.11 [95% CI 0.90–1.35]; p = 0.33; I^2^ = 21% |
| Jung 2019^78^ | Anaemia during pregnancy:  Five cohort studies; OR 2.87 [95% CI 0.59–14.10); I^2^ = 73.6; p value = 0.004 |
| Karami 2024^79^ | Maternal age:  Two studies reported association, while 3 studies reported insufficient evidence of association between maternal age and neonatal mortality.  Place of residence:  Two studies reported association, while 2 studies reported insufficient evidence of association between place of residence and neonatal mortality.  Mode of delivery:  Three studies reported association while 2 studies reported insufficient evidence of association between mode f delivery and neonatal mortality.  Interpregnancy interval:  Two studies reported the association between pregnancy interval and neonatal mortality  Multiple pregnancies:  Two studies reported association between multiple pregnancies and neonatal mortality.  Neonate sex:  Four studies showed insufficient evidence between neonate sex and child mortality  Preterm birth:  Three studies reported the association between preterm birth and neonatal mortality.  Birth order:  Two studies reported association, while 2 studies reported insufficient evidence of association between birth order and neonatal mortality  Birth weight.  Six studies reported the association of birth weight with neonatal mortality. |
| Keag 2018^80^ | Caesarean section delivery:  Five studies involving 10, 275 127 participants reported that caesarean section delivery had no association with Neonatal mortality, OR 1.01 95% CI 0.98–1.05], I^2^=0% |
| Khan 2015^81^ | Late breastfeeding initiation:  Neonates who started to breastfeed after the first hour of birth (Compared to those who started breastfeeding within 1 hour), two moderate quality studies including 34,609 participants, OR 2.02 [95% CI 1.40–2.93), I^2^=64%. Neonates who started to breastfeed after the first 24 hours of birth (Compared to those who started breastfeeding within 24 hours), 3 moderate quality studies (45,073 participants), OR 1.73 [95% CI 1.42–2.11], I^2^=0%. |
| Kozuki 2013^82^ | Interpregnancy interval:  The review reported no significant association between both short and long birth interval and neonatal mortality compared to 36-<60 months birth interval. <18 months, OR 1.49 (0.93-2.37). 18-<24 months, OR 1.07 [95% CI 0.52–2.22]; 24-<36 months; OR 0.95 [95% CI 0.62–1.47]; ≥60 months, OR 1.01 [95% CI 0.68–1.49]. |
| Lean 2017^83^ | Advanced maternal age (>35 years):  Twenty-seven studies including, 13,245,799 births, OR 1.48 [95% CI 1.30–1.67]; I^2^= 82.6% |
| Li 2021^84^ | Hypertensive disorder of pregnancy:  33 studies 4,027,092 participants reported, OR 1.55 [95% CI 1.18–2.02]; I^2^ = 95.1%;p-value = 0.000 |
| Maraka 2016^85^ | Sub-clinical hypothyroidism:  Compared with euthyroid pregnant women, pregnant women with sub-clinical hypothyroidism had a higher risk of neonatal death 6 studies, RR 2.58 (1.41, 4.73); I^2^ = 0%. |
| Mengistu 2020^86^ | Severe maternal morbidity:  13 studies involving 6,699,953 reported, OR 4.02 [95% CI 2.45, 6.59]; I^2^ = 89%.  Haemorrhagic disorder:  Seven studies including 1,791,1166 participants, OR 7.33 [95% CI 3.06–17.53]; I^2^ = 94%.  Hypertensive disorders:  Five studies including 1,791,426 participants, OR 3.00 [95% CI 1.78–5.07); I^2^ =39%.  Hepatic disorders:  Two studies including 1,219,792 participants, OR 0.92 [95% CI 0.47–1.79]; I^2^ = 0% |
| Muglu 2019^87^ | Gestational age:  The risk of neonatal death was unchanged for births between 38 and 41 weeks of gestation; the risk increased beyond 41 weeks, 5 studies included 1,127,117 pregnancies. RR 1.87 [95% CI 1.07–2.86]; p = 0.012). Sensitivity analysis performed by only including studies on singleton pregnancies uncomplicated by congenital foetal malformations, and only high-quality studies, showed a similar pattern, with increased risks observed for births beyond 42 weeks compared to the previous week. Publication bias and heterogeneity were not reported. |
| Murphy 2013^29^ | Maternal asthma  (Compared to mothers without asthma); 6 studies (2 prospective and 4 retrospective cohort), including, 57,061 participants, RR 1.49 [95% CI 1.11–2.00]; no heterogeneity; I^2^ = 0%; P = 0.67. |
| Nguyen 2019^88^ | Maternal death:  For children whose mother died when they were ≤ 0–42 days, the RR 11.3 [95% CI 5.9–21.8]; I^2^ = 86.7%; p = 0.001, pooled estimate from 4 studies and 452,699 participants. |
| Pineles 2015^89^ | Maternal smoking exposure during pregnancy:  Any active smoking; 28 studies involving 4,557,464 participants, RR 1.22 [95% CI 1.14–1.30], I^2^ = 39%, P < 0.05, no publication bias. Smoking (1-10 cigarettes/day): 10 studies RR 1.06 (0.90, 1.26). Smoking (11-20 cigarettes/day: 4 studies RR 1.30 [95% CI 1.00–1.68]. Smoking (>20 cigarettes/day): 5 studies RR 1.31 [95% CI 1.11–1.55]. Second-hand smoke exposure RR 0.98 [95% CI 0.81-1.18). The risk was computed from single pregnancy, publication bias was assessed only for any active smoking, I2 = 39%, P < 0.05. |
| Quansah 2015^90^ | Arsenic exposure:  Five studies examined neonatal mortality OR was 1.51 [95% CI 1.28–1.78]; I^2^ = 24.1%, p = 0.260. The direction of association did not change in studies applying biomarkers/individual arsenic data and in studies adjusting for adequate potential confounders. Evidence of publication bias was observed in the funnel plot. The trim and fill method imputed two studies, and the strength of association was reduced marginally. |
| Rahman 2016^91^ | Maternal anaemia:  Two studies (1,422 participants), RR 2.72 [95% CI 1.19–6.25]; I^2^ = 0.0%; no publication bias |
| Rahman 2020^92^ | Maternal anaemia:  Two studies involving 160,585, non-significant associations were seen with neonatal mortality, OR 1.80 [95% CI 0.90–27.77], I^2^ = 37% |
| Rathore 2022^93^ | Dengue virus infection:  Four studies 2 prospective and 2 retrospective cohort studies with a total of 4161 participants reported, OR 3.03 [95% CI 1.17–7.83]; I^2^ = 0%), low grade quality. |
| Robjin 2024^94^ | Maternal asthma:  women with asthma compared with those born to women without asthma, including eight cohort studies, RR 1.33 [95% CI 0.95–1.84] and no heterogeneity between studies (I^2^ = 22%, P = 0.44). |
| Shah 2011^95^ | Indigenous status (Aboriginal/non-aboriginal):  Twelve studies involving 32,799,670 participants, unadjusted, 12 studies OR 1.76 [95% CI 1.45–2.13]. 5 studies, no significant association, AOR 1.18 [95% CI 0.98–1.42]. |
| Shen 2023^96^ | Hepatitis C infection:  Compared to non-infected mother, Hepatitis C infection; 2 studies with 1,448,480 participants reported, RR 1.54 [95% CI 1.18–2.02]; I^2^ = 0.0%. |
| Smith 2017^97^ | Breastfeeding initiation:  Compared to infants started breast feeding within an hour, delayed breastfeeding initiation (2-23 hours); studies including 136,047 infants, RR 1.33 [95% CI 1.13–1.56]; I^2^ = 0.0%; p=0.7. Compared to infants started breast feeding within an hour, delayed breastfeeding initiation (for 24 hours or more); RR 2.19 [95% CI 1.73–2.77]; I^2^ = 32.5%, p = 0.2. Compared to infants started breast feeding within 24 hours, delayed breastfeeding initiation (for 24 hours or more); RR 1.70 [95% CI 1.44–2.01]; I^2^ = 41,2%. |
| Tang 2025^98^ | Interpregnancy weight gain (Two body mass index units or a body mass index class change to a higher category):  Including four cohort studies (982,878 participants) OR = 1.39 [95% CI 1.20–2.70]; I^2^ = 0% |
| Tekelab 2019^99^ | Antenatal care:  Having at least one antenatal care utilization (compared to women who had no antenatal care follow-up); OR 0.61 [95% CI 0.43–0.86]; I^2^ = 92.0%, p = 0.000; no publication bias. |
| Thomson 2021^100^ | Occupational status:  Women who had Intermediate occupations (compared to highest occupation), 2 studies including 7,561,468, OR 1.21 [95% CI 1.05–1.39]; I^2^ = 93.7%. women who had lowest occupations (manual, unskilled, never worked/ unemployed) (compared to highest occupation), 3 studies including 9,218,373; OR 1.39 [95% CI 1.22–1.57]; I^2^=97.1%. |
| Tiruye 2022^101^ | Antenatal care uptake:  (compared to women who had no antenatal care follow-up); OR 0.58 [95% CI 0.47–0.71] I^2^ = 89%; p<0.001; no publication bias. |
| Tiruye 2023^102^ | Antenatal care uptake:  Having at least one antenatal care utilization (compared to women who had no antenatal care follow-up); OR 0.59 [95% CI 0.45–0.77]; I^2^ = 95.3%; p<0.001; no publication bias. |
| Tolossa 2020^103^ | Antenatal care uptake:  (compared to women who had no antenatal care follow-up); OR 0.35 [95% CI 0.24–0.51]; I^2^=90.9%; p = 0.000; no publication bias. |
| Tura 2013^104^ | Health facility delivery:  Neonates who were delivered at health facility (compared those who were delivered at home); RR 0.40 [95% CI 0.39–0.42]; I^2^ = 97.0%; Egger’s test p = 0.003, after trim and fill analysis RR 0.71 [95% CI 0.54–0.87]. |
| Veloso 2018^105^ | Maternal education:  Complete elementary school did not show a significant association with neonatal mortality, 2 studies, OR 1.85 [95% CI 0.417–8.241]; I^2^= 87.4%; p = 0.005.  Incomplete elementary school, 2 studies, OR 1.727 (0.466-6.400), I^2^= 84.8%, p = 0.010).  Maternal marital status: women who had no partner (compared to married women), OR 2.236 (1.630-3.068), I^2^= 0.0%, p = 0.344).  Maternal age; Neonates of aged 35 years or older, OR 1.57 [95% CI 1.14–2.15]; I^2^ = 0.0%, p = 0.882. Neonate sex; Male newborn, OR 1.59 [95% CI 1.19–2.12]; I^2^ = 0.0%; p = 0.362.  History of still birth:  Two studies, OR 2.090 [95% CI 0.76–5.73]; I^2^ = 90.6%; p = 0.001.  Multiple pregnancy: OR 3.36 [95% CI 1.61–7.01) I^2^ = 48.7%; p = 0.163.  Absence of prenatal care: two studies, OR 6.80 [95% CI 1.53–30.15]; I^2^ = 78.8%; p = 0.030).  Complication during pregnancy; OR 6.961 (4.979-9.733), I^2^= 0.0%, p = 0.381.  Birth weight (after sensitivity analysis); 1500 and 2499 gm, OR 5.42 [95% CI 4.13–7.13]; I^2^= 0.0%; p = 0.902, lower than 1500 gm, OR 39.99 [95% CI 29.03–55.11]; I^2^ = 0.0%; p = 0.633) and lower than 2500 gm, OR 15.52 [95% CI 10.00–24,09], I^2^ = 0.0%; p = 0.684.  Congenital malformation: OR 14.02 [10.85–18.13]; I^2^= 0.0%; p = 0.402.  The APGAR score less than seven at 5th minute; OR 12.40 (9.54–16.10]; I^2^ = 0.0%; p = 0.417.  Gestational age (after sensitivity analysis); OR 7.13 [95% CI 4.33–11.74]; I^2^ = 0.0%; p = 0.706.  Caesarean section delivery (after sensitivity analysis); OR 1.55 [95% CI 1.20–2.00]; I^2^= 0.0%; p = 0.480. |
| Wedi 2016^106^ | Maternal HIV infection:  Neonatal mortality: maternal HIV infection, three prospective cohort studies, RR 1.68 (0.45-6.29), I^2^=89.9%. One retrospective cohort study, RR 2.00 (0.61-6.54) |
| Weightman 2012^107^ | Area deprivation:  Highest vs lowest area deprivation quintiles: two studies, OR 1.61 [95% CI 1.08–2.39]; heterogeneity p<0.001.  Social class:  Lowest vs highest social class; two studies, OR 1.42 [95% CI 1.33–1.51]; Heterogeneity p<0.001. |
| Wondemagegn 2018^108^ | Complete antenatal care uptake:  Eighteen studies, RR 0.66 [95% CI 0.54–0.80]; I^2^ = 80.5%; Egger’s test p = 0.48 |
| Wulandari 2021^109^ | Maternal Obesity:  Obese pregnant women, 8 studies (4 cohort and 4 cross-sectional) including 464,989 participants. 4 cohort studies involving 176,972 participants, OR 1.52 [95% CI 1.17–1.99]; I^2^ =0.0%; funnel plot showed publication bias. 4 cross-sectional studies involving 288,017, OR 1.44 [95% CI 1.26–1.64]; I^2^ = 0.0%, no publication bias. |
| Xie 2021^110^ | Maternal psoriasis:  Neonates of women with psoriasis (compared to normal women), 3 studies with approximately 1,756,215 participants found non-significant association, OR 1.13 [95% CI 0.90–1.43], I^2^ = 0%.No evidence of publication bias. |
| Yarandi 2021^112^ | Mild gestational diabetes mellitus:  Three studies (1889 pregnant women), RR 1.0 [95% CI 0.3–2.9], I^2^ = 7.5%; p = 0.339; no publication bias; no major single study effect. |
| Yang 2022^111^ | COVID 19 pandemic:  10 studies of 88 131 neonates during the pandemic period did not show any difference in neonatal mortality between the pandemic and pre-pandemic periods, OR 1.17 [0.81–1.70], I^2^ = 94% |
| Young 2023^113^ | Maternal anaemia:  Maternal haemoglobin (<110g/l); 10 studies 1,197,735 with participants, OR 1.25 [95% CI 1.16–1.34]. Haemoglobin (<100g/l); OR 1.37 [95% CI 1.26–1.48]. Haemoglobin (<90g/l); OR 1.46 [95% CI 1.30–1.63]. Haemoglobin (<80g/l); 1.62 (1.37–1.90). Haemoglobin (<70g/l); RR 1.83 [95% CI 1.52–2.19]. |
| Younger 2022^114^ | household air pollution from unclean cooking fuels:  The association between unclean cooking fuel and neonatal mortality was reported in six studies: as increased odds ratio in four studies, increased hazard ratio in one study and a risk ratio in a RCT study. Cooking with polluting fuels was significantly associated with an increased risk neonatal mortality, OR 1.38 [95% CI 1.14–1.67] compared to households cooking with clean fuels. |
| Yu 2017^115^ | Pre-gestational diabetes mellitus:  Nineteen studies with 7,634,750 participants, OR 2.26 [95% CI 1.74–2.95]; p(Q) = 0.001. Only cohort studies, 7 studies, OR 2.46 [1.99–3.05], p(Q) = 0.70. Non-cohort studies, 12 studies, OR 2.28 [95% CI 1.46–3.57]; p (Q)<0.0001. Type 1 diabetes mellitus, 8 studies OR 2.73 [95% CI 2.13–3.49), p (Q) = 0.44. Type 2 diabetes mellitus, 3 studies, 3.57 [95% CI 1.87– 6.80], p (Q)<0.37. |
| CI = confidence interval. I^2^ = Heterogeneity statistics. OR = odds ratio. Q = Cochran’s Q test. RR = relative risk. | |

# **Supplementary Table S9: Summary of evidence of meta-analyses on neonatal mortality**

| Risk factors | Meta-analysis | No of primary studies; study design | Total participants | Evidence of significant publication bias | I^2^ (%) | RR (95% CI) | Consistency, confidence |
| --- | --- | --- | --- | --- | --- | --- | --- |
| Socio-demographic, economic, environmental factors | | | | | | | |
| Advanced maternal age (≥35 years) | Lean 2017^83^ | 27; 15 cohort, 12 case-control | 13,245,799 | NA | 82.6 | 1.48 (1.30–1.67) | ++, Pe |
|  | Veloso 2019^105^ | 2; all cohort | 56,604 | NA | 0.0 | 1.57 (1.14–2.15) |  |
| Single motherhood | Veloso 2019^105^ | 2; 1 cohort, 1 case-control | 24,788 | NA | 0.0 | 2.24 (1.63–3.07) | ++, Ls |
| Rural residence | Aynalem 2021^55^ | 6; 3 cohort, 1 case-control, 2 cross-sectional | NA | No | 92.2 | 1.93 (1.13–2.73) | +, Ls |
| Maternal education (Each 1 additional 1 yr ) | Balaj 2021^56^ | 300*: NA | NA | NA | NA | 0.985 (0.984–0.987) | NG |
| Maternal education (secondary education vs. no) | Balaj 2021^56^ | 300*; NA | NA | NA | NA | 0.836 (0.820–0.858) | NG |
| Paternal education ( Each 1 additional 1 yr) | Balaj 2021^56^ | 300*; NA | NA | NA | NA | 0.989 (0.988–0.992) | NG |
| Paternal education (secondary education vs. no) | Balaj 2021^56^ | 300*; NA | NA | NA | NA | 0.877 (0.864–0.908) | NG |
| Maternal education (tertiary vs. primary) | Veloso 2018^105^ | 2; all cohort | 56,604 | NA | 87.4 | 0.54 (0.12–2.38) | --, Ls |
| Maternal education (tertiary vs. incomplete primary) | Veloso 2018^105^ | 2; all cohort | 56,604 | NA | 84.8 | 0.57 (0.15–2.13) | --, Ls |
| Occupation (Intermediate vs Higher) | Thomson 2021^100^ | 2; all cohort | 7,561,468 | NA | 93.7 | 1.21 (1.05–1.39) | +, Ls |
| Occupation (lower vs higher) | Thomson 2021^100^ | 3; all cohort | 9,218,373 | NA | 82.1 | 1.58 (1.44–1.74) | ++, Pe |
| Aboriginal | Shah 2011^95^ | 5; all cohort | NA | NA | NA | 1.18 (0.98–1.42) | NG |
| Immigrant | Behboudi-Gandevani 2022^57^ | 14: NA | 22,204,603 | No | 93.6 | 1.06 (1.00–1.13) | NG |
| Social class (lowest vs highest social class) | Weightman 2012^116^ | 3; all cohort | 15,899,020 | NA | NA | 1.42 (1.33–1.51) | +, Ls |
| Area deprivation (highest vs lowest area deprivation quintiles) | Weightman 2012^116^ | 3; all cohort | >843,769 | NA | NA | 1.61 (1.08–2.39) | +, Ls |
| Poor household | Jahan 2008^76^ | 3; 1 case-control, 2 cross-sectional | NA | NA | NA | 1.26 (1.12–1.42) | +, Lnc |
| Arsenic exposure | Quansah 2015^90^ | 5; 3 cohort, 2 cross-sectional | >39,759 | Yes | 24.0 | 1.51 (1.28–1.78) | ++, Pe |
| Maternal behavioural and reproductive health-related factors | | | | | | | |
| *Maternal smoking (Ref_non-smoker)* |  |  |  |  | |  |  |
| Any active cigarette smoking | Pineles 2015^89^ | 28; 26 cohort, 3 case-control | 4,557,464 | No | 39.0 | 1.22 (1.14–1.30) | NG |
| Smoking (1-10 cigarettes/day) | Pineles 2015^89^ | 10; NA | NA | NA | NA | 1.06 (0.90–1.26) | NG |
| Smoking (11-20 cigarettes/day | Pineles 2015^89^ | 4; NA | NA | NA | NA | 1.30 (1.00–1.68) | NG |
| Smoking (>20 cigarettes/day) | Pineles 2015^89^ | 5; NA | NA | NA | NA | 1.31 (1.11–1.55) | NG |
| Former smoking | Pineles 2015^89^ | 4; NA | NA | NA | NA | 0.98 (0.81–1.18) | NG |
| Prenatal opioid exposure | Graeve 2021^68^ | 14; 12 cohort, 2 case-control | 522,073 | NA | 73.0 | 4.05 (2.12–7.72) | ++, Pe |
|  | Hulse 1998^74^ | 6; 4 cohort, | 120,227 | NA | NA | 2.73 (1.41–5.28) |  |
| *Maternal BMI (ref-(18.5-24.9 kg/m2)* |  |  |  |  |  |  |  |
| Maternal underweight (<18.5 kg/m2) | Huo 2021^75^ | 12; all cohort | 3,848,782 | No | 16.0 | 1.08 (0.93–1.26) | 0, Pe |
| Maternal overweight (25.0-29.9 kg/m2) | Huo 2021^75^ | 12; all cohort | 3,848,782 | No | 62.4 | 1.23 (1.08–1.39) | +, Pe |
| Maternal obesity (≥30 kg/m2) | Huo 2021^75^ | 13; all cohort | 3,848,782 | No | 75.6 | 1.55 (1.28–1.67) | ++, Pe |
|  | Wulandari 2021^109^ | 8; 4 cohort, 4 cross-sectional | 464,989 | Yes | 0.0 | 1.45 (1.29–1.64) |  |
| Maternal BMI (5-unit increase) | Aune 2014^54^ | 12; all cohort | 3,321,555 | No | 78.5 | 1.15 (1.07–1.23) | ?, Ls |
| Interpregnancy weight gain | Tang 2025^989^ | 4; all cohort | 982,878 | NA | 0.0 | 1.39 (1.20–2.70) | +, Ls |
| *Interpregnancy interval* |  |  |  |  |  |  |  |
| Short interpregnancy interval  (Ref-≥ 24 months) | Hassen 2024^70^ | 6; all cross-sectional | 105,291 | Yes | 95.2 | 1.78 (1.25–2.55) | ++, Ls |
|  | Islam 2022^71^ | 17; 1 cohort. 16 cross-sectional | >279,345 | Yes | 72.6 | 1.85 (1.68–2.04) |  |
| Interpregnancy interval (<18 Vs 36-<60 months) | Kozuki 2013^82^ | 5; all cohort | 19,240 | NA | NA | 1.49 (0.93–2.37) | ?, Ls |
| Interpregnancy interval (18-<24 Vs 36-<60 months) | Kozuki 2013^82^ | 5; all cohort | 19,240 | NA | NA | 1.07 (0.52–2.22) | ?, Ls |
| Interpregnancy interval (24-<36 Vs 36-<60 months) | Kozuki 2013^82^ | 5; all cohort | 19,240 | NA | NA | 0.95 (0.62–1.47) | 00, Pe |
| Interpregnancy interval (≥60 Vs 36-<60 months) | Kozuki 2013^82^ | 5; all cohort | 19,240 | NA | NA | 1.01 (0.68–1.49) | 00, Pe |
| History of CS delivery | Keag 2018^80^ | 5; all cohort | 10,275,127 | NA | 0.0 | 1.01 (0.98–1.05) | 00, Pe |
| History of stillbirth | Deng 2024^64^ | 7; all cohort | 362,300 | NA | 47.0 | 4.23 (2.64–6.72) | ++, Pe |
| ANC uptake (Yes vs No) | Tekelab 2019^99^ | 12; 5 cohort, 2 case-control, 5 cross-sectional | 79,990 | No | 92.0 | 0.62 (0.44–0.89) | --, Pe |
|  | Tiruye 2022^101^ | 27; 12 cohort, 10 cross-sectional, 5 case- control | 85,530 | No | 89.0 | 0.58 (0.47–0.71) |  |
|  | Tiruye 2023^102^ | 28; 13 cohort, 7 case-control, 8 cross-sectional | 61,730 | No | 95.3 | 0.59 (0.45–0.77) |  |
|  | Tolossa 2020^103^ | 14; 6 cohort, 5 case-control, 3 -cross-sectional | 23, 932 | No | 90.9 | 0.39 (0.26–0.54) |  |
|  | Veloso 2019^105^ | 2: 1 cohort, 1 case-control | 32,734 | NA | 78.8 | 0.15 (0.03–0.65) |  |
|  | Wondemagegn 2018^108^ | 18; 1 community trial, 4 cohort, 4 case-control, 9 cross-sectional | 156,512 | No | 80.5 | 0.67 (0.55–0.80) |  |
| Complete ANC uptake | Belachew 2020^58^ | 3; 1 cohort, 1 cross-sectional, 1 unknown | NA | NA | 84.1 | 0.57 (0.41–0.77) | --, Ls |
|  | Helmyati 2022^73^ | 4; 1 cohort, 3 case-control | 12,032 | No | 93.0 | 0.42 (0.17–0.99) |  |
| Health facility delivery | Chaka 2019^61^ | 19; 1 community trial, 4 cohort, 8 case-control, 6 cross sectional | 1,045,562 | Yes | 84.5 | 0.48 (0.38–0.58) | --, Pe |
|  | Tura 2013^104^ | 19; 1 community trial, 5 cohort, 4 case-control, 9 cross-sectional | 1,606,805 | Yes | 97.0 | 0.71 (0.54–0.87) |  |
| Pregnancy and neonatal outcomes | | | | | | | |
| Complication during pregnancy | Veloso 2018^105^ | 2; 1 cohort, 1 case-control | 24,788 | NA | 0.0 | 6.96 (4.98–9.73) | ++, Ls |
| Severe maternal morbidity | Mengistu 2020^86^ | 13; 9 cohort, 4 case-control | 6,699,953 | NA | 89.0 | 3.46 (2.27–5.64) | ++, Pe |
| Hyperemesis gravidarum | Jansen 2023^77^ | 5; all cohort | 3,973,154 | NA | 21.0 | 1.11 (0.90–1.35) | ?, LS |
| Mild gestational diabetes mellitus | Yarandi 2021^112^ | 2; NA | 1889 | No | 7.5 | 1.00 (0.30–2.90) | NG |
| Hypertensive disorder of pregnancy | Li 2021^84^ | 33: all cohort | 4,027,092 | No | 95.1 | 1.55 (1.18–2.02) | ++, Pe |
|  | Mengistu 2020^86^ | 5; 3 cohort, 2 case-control | 1,791,426 | No | 39.0 | 2.87 (1.75–4.65) |  |
| Haemorrhagic disorder during pregnancy or childbirth | Mengistu 2020^86^ | 5; 4 cohort, 1 case-control | 1,791,116 | NA | 94.0 | 7.20 (3.04–16.75) | ++, Pe |
| CS delivery | Veloso 2019^105^ | 2; 1 cohort, 1 case-control | 171,036 | NA | 89.7% | 1.23 (0.69–2.21) | ?, Lnc |
| Maternal death | Nguyen 2019^117^ | 4; all cohort | 452,699 | NA | 86.7 | 11.30 (5.90–21.80) | ++, Pe |
| Male neonate | Veloso 2019^105^ | 2; 1 cohort, 1 case-control | 24,725 | NA | 0.0 | 1.59 (1.19– 2.12) | ++, Ls |
| Gestational age (41^+0-6^ vs. 40^+0-6^ weeks) | Muglu 2019^87^ | 5; all cohort | 7,194,423 | No | NA | 1.87 (1.07–2.86) | NG |
| Gestational age (42^+0-6^ Vs. 41^+0-6^ weeks) | Muglu 2019^87^ | 4; all cohort | NA | No | NA | 1.32 (0.20–3.38) | NG |
| Preterm birth | Aynalem 2021^55^ | 7; 4 cohort, 1 case-control, 2 cross-sectional | NA | No | 38.0 | 1.32 (1.07–1.58) | ++, Pe |
|  | Desta 2021^65^ | 9; 6 cohort, 3 cross-sectional | 10,149 | No | 95.7 | 3.16 (1.57–6.34) |  |
|  | Veloso 2019^105^ | 6; 2 cohort, 4 case-control | 155,846 | NA | 0.0 | 7.13 (4.33–11.74) |  |
| Apgar score < 7 at the 5th minute | Veloso 2019^105^ | 3; all cohort | 178,615 | NA | 0.0 | 12.40 (9.54–16.10) | ++, LS |
| *Late breastfeeding initiation* |  |  |  |  |  |  |  |
| ≥24 hrs Vs. within 24hrs | Debes 2013^63^ | 3; all cohort | 44, 249 | NA | NA | 1.58 (1.26–2.50) | ++, Pe |
|  | Khan 2015^81^ | 3; all cohort | 45,073 | NA | 64.0 | 1.73 (1.42–2.11) |  |
|  | Smith 2017^97^ | 6; all cohort | 142,729 | NA | 41.2 | 1.70 (1.44–2.01) |  |
| 2-23hrs Vs within 1 hr | Smith 2017^97^ | 3; all cohort | 136,047 | NA | 0.0 | 1.33 (1.13–1.56) | ?, Ls |
| ≥24hrs Vs within 1 hr | Smith 2017^97^ | 3; all cohort | 136,047 | NA | 32.5 | 2.19 (1.73–2.77) | ++, Ls |
| Exclusively breastfed vs. partial breastfeeding | Smith 2017^97^ | 2; all cohort | 10,423 | NA | NA | 0.27 (0.15–0.49) | --, Ls |
| Low birth weight < 2500g | Veloso 2019^105^ | 7; 2 cohort, 5 case-control | 18,239 | NA | 0.0 | 15.52 (10.00–24.09) | ++, Pe |
| Low birth weight (1500-2499) g | Veloso 2019^105^ | 3; all cohort | 195,011 | NA | 0.0 | 5.42 (4.13–7.13) | ++, Pe |
| Low birth weight < 1500g | Veloso 2019^105^ | 3; all cohort | 195,011 | NA | 0.0 | 40.00 (29.03–55.11) | ++, Pe |
| Maternal infectious and chronic medical conditions | | | | | | | |
| Anaemia during pregnancy (<11 g/dl) | Jung 2019^78^ | 5; all cohort | NA | No | 73.6 | 2.87 (0.59–14.10) | ++, Pe |
|  | Rahman 2016^91^ | 2; all cohort | 1242 | No | 0 | 2.72 (1.19–6.25) |  |
|  | Rahman 2020^92^ | 2; all cohort | 160,585 | NA | 37.0 | 1.80 (0.90–27.77) |  |
|  | Young 2023^113^ | 10; 8 cohort, 1 case-control, 1 cross-sectional | 1,197,735 | NA | NA | 1.25 (1.16–1.34) |  |
| Dengue virus infection during pregnancy | Rathore 2022^93^ | 4; all cohort | 4161 | No | 0.0 | 3.03 (1.17–7.83) | +, Ls |
| COVID 19 pandemic | Chmielewska 2021^62^ | 3; NA | 93,111 | NA | 85.0 | 1·01 (0·38–2.67) | NG |
|  | Yang 2022^111^ | 10; NA | 88, 131 | NA | 94.0 | 1.17 (0.81–1.70) |  |
| Chlamydia trachomatis | He 2020^72^ | 3; all cohort | NA | NA | 27.5 | 0.99 (0.49–2.02) | NG |
| Hepatitis B virus infection | Afraie 2023^52^ | 6; all cohort | 182,421 | No | 0.0 | 0.83 (0.67–1.03) | 00, Pe |
| Hepatitis C infection | Shen 2023^96^ | 2; all cohort | 1,448,480 | NA | 0.0 | 1.54 (1.18–2.02) | ++, Ls |
| HIV infection | Brocklehurst 1998^118^ | 3; all cohort | 1,517 | NA | NA | 1.10 (0.63–1.93) | ?, Ls |
|  | Wedi 2016^106^ | 3; all cohort | 2,297 | NA | 89.9 | 1.68 (0.45–6.29) |  |
| Sub-clinical hypothyroidism | Maraka 2016^85^ | 6; all cohort | NA | NA | 0.0 | 2.58 (1.41–4.73) | NG |
| Psoriasis | Xie 2021^110^ | 3; 2 cohort, 1 case-control | 1,756,215 | No | 0.0 | 1.13 (0.90–1.43) | 0, Ls |
| Asthma | Robjin 2024^94^ | 8; all cohort | 241,715 | NA | 22.0 | 1.33 (0.95–1.84) | ?, Ls |
| Sickle-cell disease | Boafor 2015^59^ | 6; 2 cohort, 4 case-control | 2,659 | NA | NA | 2.71 (1.41–5.22) | +, Ls |
| Hepatic disorder | Mengistu 2020^86^ | 2; 1 cohort, 1 case-control | 1,219,792 | NA | 0.0 | 0.92 (0.47–1.79) | ?, Lnc |
| Pre-gestational DM | Yu 2017^115^ | 19; NA | 7,634,750 | No | 59.0 | 2.26 (1.74–2.95) | NG |
| Type 1 DM | Yu 2017^115^ | 8; NA | NA | NA | NA | 2.73 (2.13–3.49) | NG |
| Type 2 DM | Yu 2017^115^ | 3; NA | NA | NA | NA | 3.57 (1.87–6.80) | NG |
| *The review did not clearly provide the number of primary studies for the association. + indicates less consistent positive association. ++ indicates consistent positive association. ? indicates unclear or contradictory direction. 0 indicates less consistent null association. 00 indicates consistent null association. - indicates less consistent negative association. -- indicates consistent negative association. ANC = antenatal care. BF = breastfeeding. CI = confidence interval. COVID-19 = Coronavirus Disease 2019. CS = caesarean section. GDM = gestational diabetes mellitus. I² = heterogeneity statistic. Lnc = limited and non-conclusive evidence. LS = Limited-suggestive evidence. NA = not available. NG = not graded. Pe = probable evidence. RR = relative risk. | | | | | | | |

# **Supplementary Table S10: Study overlaps in the included meta-analyses**

| Meta-analyses reported similar factors | Total number of times studies appeared in the meta-analyses (N) | Number of indexed primary study (r) | Number of reviews (c) | Corrected covered area (CCA in %) | Degree of overlap |
| --- | --- | --- | --- | --- | --- |
| Meta-analyses on advanced maternal age (<35 yrs) | 29 | 29 | 2 | 0.0 | None |
| Meta-analyses on prenatal opioid exposure | 20 | 17 | 2 | 17.6 | Very high |
| Meta-analyses maternal obesity | 21 | 19 | 2 | 10.5 | High |
| Meta-analyses on ANC uptake | 101 | 58 | 5 | 18.5 | Very high |
| Meta-analyses on complete ANC uptake | 7 | 7 | 2 | 0.0 | None |
| Meta-analyses on health facility delivery | 38 | 31 | 2 | 22,6 | Very high |
| Meta-analyses on anaemia during pregnancy | 19 | 15 | 4 | 8.9 | Moderate |
| Meta-analyses on HIV-infection | 6 | 4 | 2 | 50.0 | Very high |
| Meta-analyses on COVID 19 pandemic | 13 | 11 | 2 | 18.2 | Very high |
| Meta-analyses on preterm birth | 22 | 18 | 3 | 11.1 | High |
| Meta-analyses on delayed breastfeeding initiation | 12 | 6 | 3 | 50.0 | Very high |

$$CCA=\frac{N-r}{rc-r}$$

Where N is the total number of primary studies include in the reviews, r is the total number of indexed primary studies, and c is the total number of reviews. The overlaps classified based on CCA score as slight (≤5%), moderate (6–10%), high (11–15%), and very high (>15%).^119^

# **Supplementary Table S11: Calculated relative risks**

| **Risk/protective factors** | **Author** | **OR (95%CI)** | **Prevalence among**  **non-exposed (%)** | **RR (95% CI)** |
| --- | --- | --- | --- | --- |
| Immigrant | Behboudi-Gandevani 2022^57^ | 1.09 (1.00–1.19) | 0.26 | 1.09 (1.00–1.13) |
| ANC uptake | Wondemagegn 2018^108^ | 0.66 (0.54–0.80) | 3.00 | 0.67 (0.55–0.80) |
| ANC uptake | Tolossa 2020^103^ | 0.35 (0.24–0.51) | 11.60 | 0.39 (0.26–0.54) |
| ANC uptake | Tekelab 2019^99^ | 0.61 (0.43–0.86) | 5.80 | 0.62 (0.44–0.89) |
| Complete ANC uptake | Helmyati 2022^73^ | 0.39 (0.15–0.99) | 13.70 | 0.42 (0.17–0.99) |
| Hyperemesis gravidarum | Jansen 2023^77^ | 1.11 (0.90–1.35) | 0.30 | 1.11 (0.90–1.35) |
| History of stillbirth | Deng 2024^64^ | 4.24 (2.65–6.79) | 0.18 | 4.23 (2.64–6.72) |
| Severe maternal morbidity | Mengistu 2020^86^ | 4.02 (2.45–6.59) | 5.30 | 3.46 (2.27–5.64) |
| Hypertensive disorder of pregnancy | Mengistu 2020^86^ | 3.00 (1.78–5.07) | 2.20 | 2.87 (1.75–4.65) |
| Haemorrhagic disorder | Mengistu 2020^86^ | 7.33 (3.06–17.53) | 0.28 | 7.20 (3.04–16.75) |
| Hepatic disorder | Mengistu 2020^86^ | 0.92 (0.47–1.79) | 0.17 | 0.92 (0.47–1.79) |
| CI = confidence interval. OR = odds ratio. RR = relative risk | | | | |

**
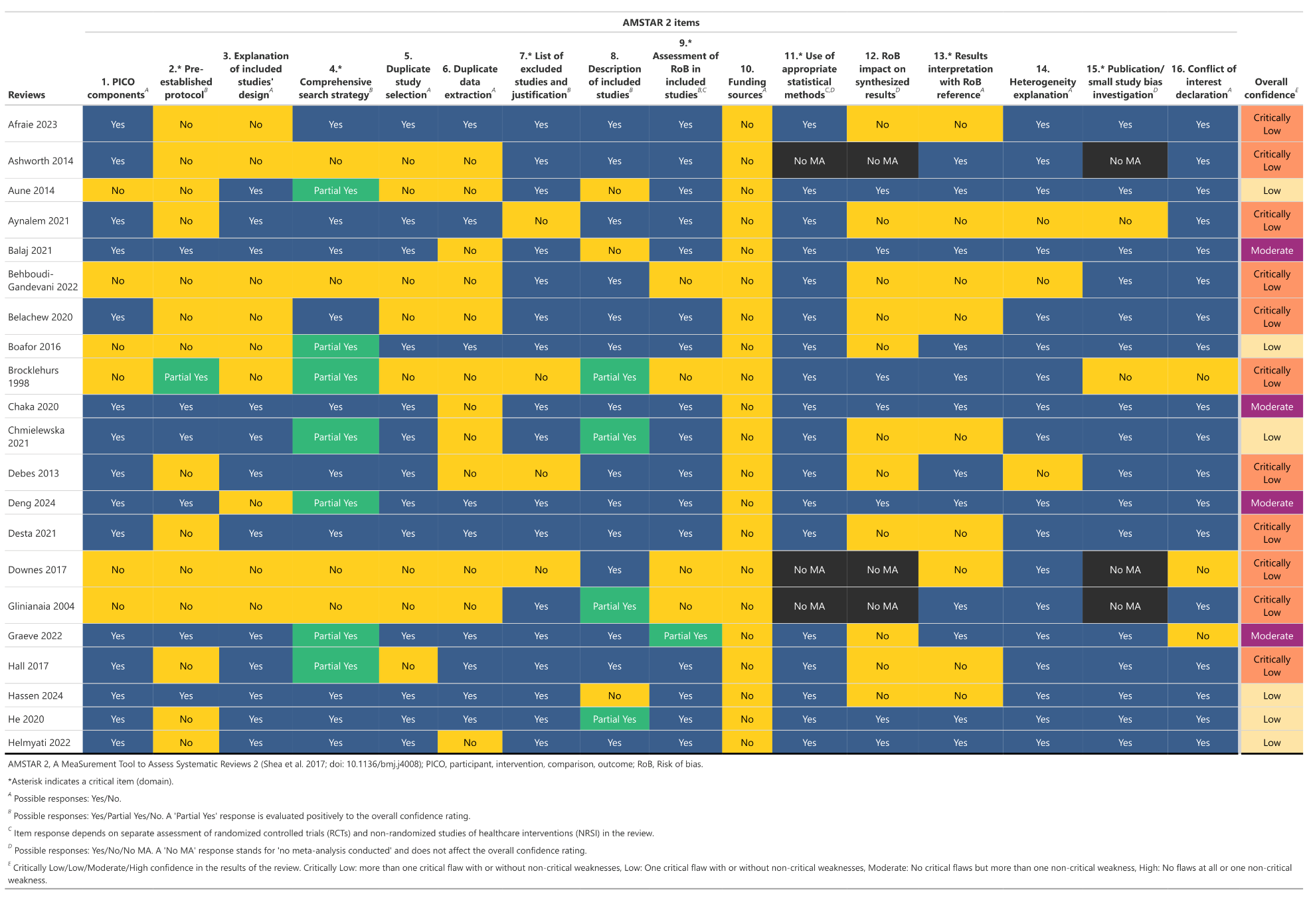
**


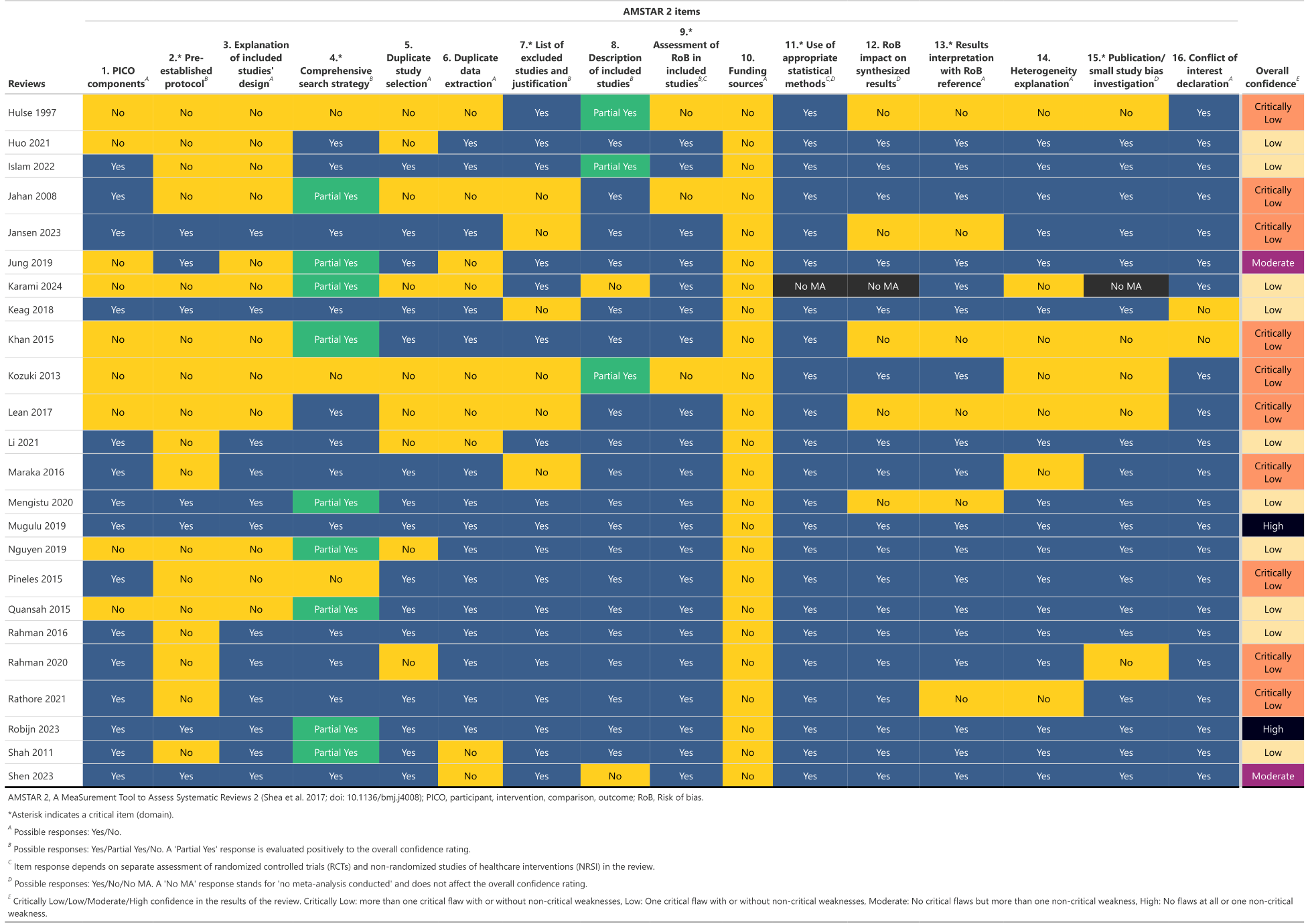

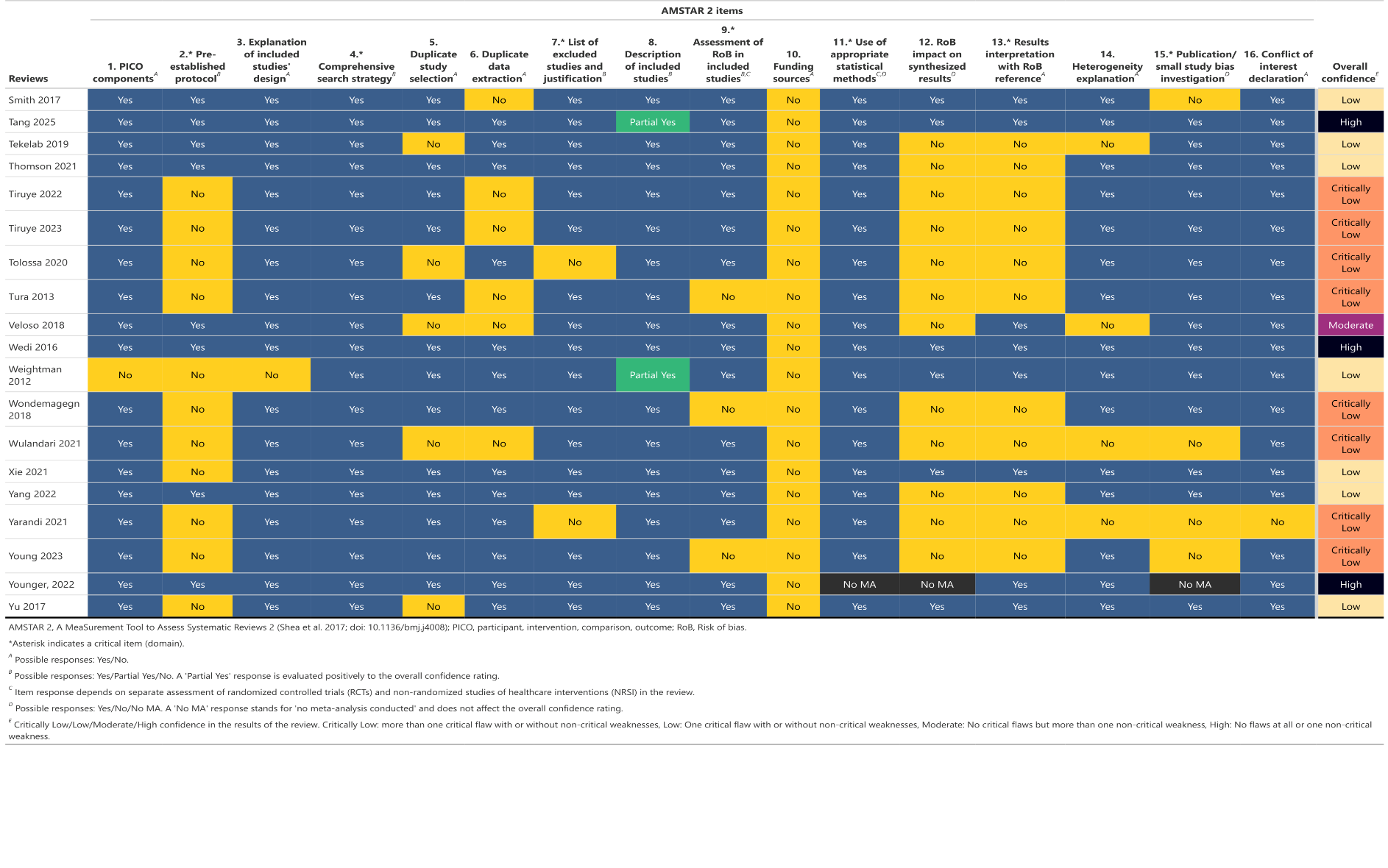


# **Supplementary Figure S1: Quality assessment using AMSTAR 2 tool**

**Modifiable**

-Maternal education

- Paternal education

- Poor wealth status

- Maternal smoking

- Prenatal opioid exposure

- Maternal body mass index

- Interpregnancy weight gain

- Interpregnancy interval

- Antenatal care uptake

- Health facility delivery

- Unintended pregnancy

- Complication during pregnancy

- Severe maternal morbidity

- Hyperemesis gravidarum

- Mild gestational diabetes mellitus

- Hypertensive disorder of pregnancy

- Haemorrhagic disorder

- Placental abruption

- Maternal death

- Anaemia during pregnancy

- Dengue virus infection

- Chlamydia trachomatis

- Hepatitis B infection

- Hepatitis C infection

- HIV infection

- Sub-clinical hypothyroidism

- Low birth weight

- Preterm birth

- Post-term birth

- Low Apgar score

- Late breastfeeding initiation

- Partial breastfeeding

**Non-modifiable**

- Advanced maternal age

- Single motherhood

- Low occupational status

- History of caesarean section

- History of stillbirth

- Multiple pregnancy

- Mode of delivery

- Psoriasis

- Asthma

- Sickle-cell disease

- Hepatic disorder

- Pregestational diabetes

- Neonatal sex

- Birth order

- Neonatal sex

- Birth order

**Non-modifiable**

- Rural residence

- Aboriginal status

- Immigrant status

**Modifiable**

- COVID-19 pandemic

- Household air pollution

- Ambient air pollution

- Waste incineration

- Arsenic exposure

System-level factors

Individual-level factors

# **Supplementary Figure S2: Risk factors of neonatal mortality based on modifiability and level of influence**

# **Supplementary Figure S3: Publication date ranges of systematic reviews**

# **References**

1. Zeiher J, Ombrellaro KJ, Perumal N, Keil T, Mensink GB, Finger JD. Correlates and determinants of cardiorespiratory fitness in adults: a systematic review. *Sports medicine-open* 2019; 5: 1-24.

2. Nyadanu SD, Dunne J, Tessema GA, et al. Prenatal exposure to ambient air pollution and adverse birth outcomes: an umbrella review of 36 systematic reviews and meta-analyses. *Environmental Pollution* 2022; 306: 119465.

3. O’Donoghue G, Kennedy A, Puggina A, et al. Socio-economic determinants of physical activity across the life course: A" DEterminants of DIet and Physical ACtivity"(DEDIPAC) umbrella literature review. *PloS one* 2018; 13(1): e0190737.

4. Shrier I, Steele R. Understanding the relationship between risks and odds ratios. *Clinical Journal of Sport Medicine* 2006; 16(2): 107-10.

5. Ahmed M, Won Y. Cross-national systematic review of neonatal mortality and postnatal newborn care: special focus on Pakistan. *International journal of environmental research and public health* 2017; 14(12): 1442.

6. Ahrens KA, Moskosky S, Nelson H, Stidd RL, Hutcheon JA. Short interpregnancy intervals and adverse perinatal outcomes in high-resource settings: An updated systematic review. *Paediatric & Perinatal Epidemiology* 2019; 33(1): O25-O47.

7. Asferie WN, Aytenew TM, Kassaw A, et al. Effect of maternal HIV infection on birth outcomes among HIV positive women in Sub Saharan Africa: a systematic review and meta-analysis. *BMC public health* 2025; 25(1): 459.

8. Bell R. Systematic review and meta-analysis: Maternal body mass index increasing above 20 is associated with increased risk of miscarriage, stillbirth, neonatal death and postneonatal death. *Evidence-Based Medicine* 2014; 19(6): 237.

9. Bitew ZW, Alemu A, Ayele EG, Jember DA, Haile MT, Worku T. Incidence density rate of neonatal mortality and predictors in sub‐Saharan Africa: a systematic review and meta‐analysis. *International journal of pediatrics* 2020; 2020(1): 3894026.

10. Brinchmann BC, Vist GE, Becher R, et al. Use of Swedish smokeless tobacco during pregnancy: A systematic review of pregnancy and early life health risk. *Addiction* 2023; 118(5): 789-803.

11. Di Toro F, Gjoka M, Di Lorenzo G, et al. Impact of COVID-19 on maternal and neonatal outcomes: a systematic review and meta-analysis. *Clinical Microbiology and Infection* 2021; 27(1): 36-46.

12. Daemi A, Ravaghi H, Jafari M. Risk factors of neonatal mortality in Iran: A systematic review. *Medical Journal of the Islamic Republic of Iran* 2019; 33(1).

13. Go MDA, Emeis C, Guise J-M, Schelonka RL. Fetal and neonatal morbidity and mortality following delivery after previous cesarean. *Clinics in perinatology* 2011; 38(2): 311-9.

14. Gissler M, Alexander S, MacFarlane A, et al. Stillbirths and infant deaths among migrants in industrialized countries. *Acta obstetricia et gynecologica Scandinavica* 2009; 88(2): 134-48.

15. Hessami K, Homayoon N, Hashemi A, Vafaei H, Kasraeian M, Asadi N. COVID-19 and maternal, fetal and neonatal mortality: a systematic review. *The journal of maternal-fetal & neonatal medicine* 2022; 35(15): 2936-41.

16. Hardee I, Wright L, McCracken C, Lawson E, Oster ME. Maternal and neonatal outcomes of pregnancies in women with congenital heart disease: a meta‐analysis. *Journal of the American Heart Association* 2021; 10(8): e017834.

17. Ho P, Quigley MA, Tatwavedi D, Britto C, Kurinczuk JJ. Neonatal and infant mortality associated with spina bifida: A systematic review and meta-analysis. *PloS one* 2021; 16(5): e0250098.

18. Hodgkin K, Joshy G, Browne J, Bartini I, Hull TH, Lokuge K. Outcomes by birth setting and caregiver for low risk women in Indonesia: a systematic literature review. *Reproductive Health* 2019; 16(1): 67.

19. Jans SM, de Jonge A, Lagro-Janssen AL. Maternal and perinatal outcomes amongst haemoglobinopathy carriers: a systematic review. *International Journal of Clinical Practice* 2010; 64(12): 1688-98.

20. Jia D, Sun F, Han S, Lu L, Sun Y, Song Q. Adverse outcomes in subsequent pregnancies in women with history of recurrent spontaneous abortion: A meta‐analysis. *Journal of Obstetrics and Gynaecology Research* 2024; 50(3): 281-97.

21. Khan KS. Maternal HIV infection increases the risk of adverse perinatal outcomes, especially infant death. *Evidence-based Obstetrics & Gynecology* 1999; 1(3): 97.

22. Kim D, Saada A. The social determinants of infant mortality and birth outcomes in Western developed nations: a cross-country systematic review. *International journal of environmental research and public health* 2013; 10(6): 2296-335.

23. Kontovazainitis C-G, Katsaras GN, Gialamprinou D, Mitsiakos G. Covid-19 vaccination and pregnancy: a systematic review of maternal and neonatal outcomes. *Journal of perinatal medicine* 2023; 51(7): 823-39.

24. Kozuki N, Lee AC, Silveira MF, et al. The associations of parity and maternal age with small-for-gestational-age, preterm, and neonatal and infant mortality: a meta-analysis. *BMC public health* 2013; 13(Supplement 3): S2.

25. Lo JO, Shaw B, Robalino S, et al. Cannabis use in pregnancy and neonatal outcomes: a systematic review and meta-analysis. *Cannabis and Cannabinoid Research* 2024; 9(2): 470-85.

26. Lalani S, Choudhry AJ, Firth B, et al. Endometriosis and adverse maternal, fetal and neonatal outcomes, a systematic review and meta-analysis. *Human Reproduction* 2018; 33(10): 1854-65.

27. Marchi J, Berg M, Dencker A, Olander E, Begley C. Risks associated with obesity in pregnancy, for the mother and baby: a systematic review of reviews. *Obesity reviews* 2015; 16(8): 621-38.

28. Martinez-Hortelano JA, Gonzalez PB, Rodriguez-Rojo IC, et al. Interpregnancy weight change and neonatal and infant outcomes: A systematic review and meta-analysis. *Annals of Epidemiology* 2024; 97: 1-10.

29. Murphy VE, Schatz M. Asthma in pregnancy: A hit for two. *European Respiratory Review* 2014; 23(131): 64-8.

30. Mersha AG, Abegaz TM, Seid MA. Maternal and perinatal outcomes of hypertensive disorders of pregnancy in Ethiopia: systematic review and meta-analysis. *BMC pregnancy and childbirth* 2019; 19: 1-12.

31. Ni W, Gao X, Su X, et al. Birth spacing and risk of adverse pregnancy and birth outcomes: A systematic review and dose-response meta-analysis. *Acta Obstetricia et Gynecologica Scandinavica* 2023; 102(12): 1618-33.

32. Pastor-Moreno G, Ruiz-Perez I, Henares-Montiel J, Petrova D. Intimate partner violence during pregnancy and risk of fetal and neonatal death: a meta-analysis with socioeconomic context indicators. *American journal of obstetrics and gynecology* 2020; 222(2): 123-33. e5.

33. Pinheiro RL, Areia AL, Mota Pinto A, Donato H. Advanced maternal age: adverse outcomes of pregnancy, a meta-analysis. *Acta medica portuguesa* 2019; 32(3): 219-26.

34. Pratiwi SR, Prasetya H, Murti B. The effect of asphyxia on neonatal death: a meta-analysis. *Journal of Maternal and Child Health* 2020; 5(4): 413-21.

35. Ramaiya A, Kiss L, Baraitser P, Mbaruku G, Hildon Z. A systematic review of risk factors for neonatal mortality in Adolescent Mother’s in Sub Saharan Africa. *BMC research notes* 2014; 7: 1-6.

36. Saccone G, Gragnano E, Ilardi B, et al. Maternal and perinatal complications according to maternal age: A systematic review and meta‐analysis. *International Journal of Gynecology & Obstetrics* 2022; 159(1): 43-55.

37. Saputri CS, Rizki AW, Flora VH, Murti B, Wulandari AN. Associations between Prematurity, Low Birth Weight, and Residence on the Risk of Newborn Death: A Meta Analysis. *Journal of Maternal &amp; Child Health (JMCH)* 2024; 9(2): 138-51.

38. Shen J, Shi M. Association between decision-to-delivery time and neonatal outcomes: a systematic review and meta-analysis. *BMC Pregnancy and Childbirth* 2024; 24: 410.

39. Shi S, Wu Y, Wu H, et al. Relationship between maternal arsenic exposure and neonatal mortality and infant mortality: A meta-analysis. *Chinese Journal of Endemiology* 2024; 43(12): 1021-6.

40. Tran NT, Taylor R, Antierens A, Staderini N. Cholera in Pregnancy: A Systematic Review and Meta-Analysis of Fetal, Neonatal, and Maternal Mortality. *PLoS ONE [Electronic Resource]* 2015; 10(7): e0132920.

41. Vallely LM, Egli-Gany D, Wand H, et al. Adverse pregnancy and neonatal outcomes associated with Neisseria gonorrhoeae: systematic review and meta-analysis. *Sexually transmitted infections* 2021; 97(2): 104-11.

42. Veenendaal MV, van Abeelen AF, Painter RC, van der Post JA, Roseboom TJ. Consequences of hyperemesis gravidarum for offspring: a systematic review and meta‐analysis. *BJOG: An International Journal of Obstetrics & Gynaecology* 2011; 118(11): 1302-13.

43. Walther F, Kuester D, Bieber A, Malzahn J, Rudiger M, Schmitt J. Are birth outcomes in low risk birth cohorts related to hospital birth volumes? A systematic review. *BMC Pregnancy and Childbirth* 2021; 21: 531.

44. Wang R, Yan W, Du M, Tao L, Liu J. The effect of influenza virus infection on pregnancy outcomes: A systematic review and meta-analysis of cohort studies. *International Journal of Infectious Diseases* 2021; 105: 567-78.

45. Wang Y, Zeng C, Chen Y, et al. Short interpregnancy interval can lead to adverse pregnancy outcomes: A meta-analysis. *Frontiers in Medicine* 2022; 9: 922053.

46. Wendt A, Gibbs CM, Peters S, Hogue CJ. Impact of increasing inter-pregnancy interval on maternal and infant health. *Paediatric and Perinatal Epidemiology* 2012; 26(SUPPL. 1): 239-58.

47. Yan SM, Zhai QF, Xing J, Li WW, Gao XC, Qiu YG. [Relationship between pesticide exposure and adverse pregnancy outcomes among famers: a meta-analysis]. *Zhonghua Lao Dong Wei Sheng Zhi Ye Bing Za Zhi/Zhonghua Laodong Weisheng Zhiyebing Zazhi/Chinese Journal of Industrial Hygiene & Occupational Diseases* 2012; 30(11): 859-62.

48. Yang W, Zu SJ, Jin Q, et al. Fetal hyperechoic kidney cohort study and a meta-analysis. *Frontiers in Genetics* 2023; 14.

49. Ye W, Luo C, Huang J, Li C, Liu Z, Liu F. Gestational diabetes mellitus and adverse pregnancy outcomes: systematic review and meta-analysis. *Bmj* 2022; 377.

50. Yitayih Y, Vanderplasschen W, Vandewalle S, Rita VD, Gilbert L. The effects of khat use during pregnancy on perinatal and maternal outcomes: a meta-analysis. *Archives of Women's Mental Health* 2023; 26(1): 11-27.

51. Zulu T, Jacobs C, Biemba G, Musonda P. Prevalence of early neonatal mortality and its predictors in sub-Saharan Africa: A Systematic review and Meta-Analysis. *medRxiv* 2024; 07.

52. Afraie M, Moradi G, Zamani K, Azami M, Moradi Y. The effect of hepatitis B virus on the risk of pregnancy outcomes: a systematic review and meta-analysis of cohort studies. *Virology Journal* 2023; 20(1): 213.

53. Ashworth DC, Elliott P, Toledano MB. Waste incineration and adverse birth and neonatal outcomes: a systematic review. *Environment international* 2014; 69: 120-32.

54. Aune D, Saugstad OD, Henriksen T, Tonstad S. Maternal body mass index and the risk of fetal death, stillbirth, and infant death: A systematic review and meta-analysis. *Jama* 2014; 311(15): 1536-46.

55. Aynalem YA, Shiferaw WS, Akalu TY, Dargie A, Assefa HK, Habtewold TD. The Magnitude of Neonatal Mortality and Its Predictors in Ethiopia: A Systematic Review and Meta-Analysis. *Int J Pediatr* 2021; 2021: 7478108.

56. Balaj M, York HW, Sripada K, et al. Parental education and inequalities in child mortality: a global systematic review and meta-analysis. *Lancet* 2021; 398(10300): 608-20.

57. Behboudi-Gandevani S, Bidhendi-Yarandi R, Panahi MH, et al. A Systematic Review and Meta-Analysis of the Risk of Stillbirth, Perinatal and Neonatal Mortality in Immigrant Women. *International journal of public health* 2022; 67: 1604479.

58. Belachew A, Tewabe T, Dessie G. Neonatal mortality and its association with antenatal care visits among live births in Ethiopia: a systematic review and meta-analysis. *Journal of Maternal-Fetal and Neonatal Medicine* 2022; 35(2): 348-55.

59. Boafor TK, Olayemi E, Galadanci N, et al. Pregnancy outcomes in women with sickle-cell disease in low and high income countries: a systematic review and meta-analysis. *BJOG: An International Journal of Obstetrics & Gynaecology* 2016; 123(5): 691-8.

60. Brocklehurst P, French R. The association between maternal HIV infection and perinatal outcome: A systematic review of the literature and meta-analysis. *British Journal of Obstetrics and Gynaecology* 1998; 105: 836-48.

61. Chaka EE, Mekurie M, Abdurahman AA, Parsaeian M, Majdzadeh R. Association between place of delivery for pregnant mothers and neonatal mortality: a systematic review and meta-analysis. *European journal of public health* 2020; 30(4): 743-8.

62. Chmielewska B, Barratt I, Townsend R, et al. Effects of the COVID-19 pandemic on maternal and perinatal outcomes: a systematic review and meta-analysis. *Lancet Global Health* 2021; 9(6): E759-E72.

63. Debes AK, Kohli A, Walker N, Edmond K, Mullany LC. Time to initiation of breastfeeding and neonatal mortality and morbidity: a systematic review. *BMC public health* 2013; 13 Suppl 3: S19.

64. Deng X, Pan B, Lai H, et al. Association of previous stillbirth with subsequent perinatal outcomes: a systematic review and meta-analysis of cohort studies. *American Journal of Obstetrics & Gynecology* 2024; 231(2): 211-22.

65. Desta M, Admas M, Yeshitila Y, et al. Effect of Preterm Birth on the Risk of Adverse Perinatal and Neonatal Outcomes in Ethiopia: A Systematic Review and Meta-Analysis. *Inquiry : a journal of medical care organization, provision and financing* 2021; 58: 469580211064125.

66. Downes KL, Grantz KL, Shenassa ED. Maternal, Labor, Delivery, and Perinatal Outcomes Associated with Placental Abruption: A Systematic Review. *American Journal of Perinatology* 2017; 34(10): 935-57.

67. Glinianaia SV, Rankin J, Bell R, Pless-Mulloli T, Howel D. Does particulate air pollution contribute to infant death? A systematic review. *Environmental Health Perspectives* 2004; 112(14): 1365-70.

68. Graeve R, Balalian AA, Richter M, et al. Infants' prenatal exposure to opioids and the association with birth outcomes: A systematic review and meta-analysis. *Paediatric and Perinatal Epidemiology* 2022; 36(1): 125-43.

69. Hall JA, Benton L, Copas A, Stephenson J. Pregnancy Intention and Pregnancy Outcome: Systematic Review and Meta-Analysis. *Maternal and child health journal* 2017; 21(3): 670-704.

70. Hassen TA, Harris ML, Shifti DM, et al. Effects of short inter-pregnancy/birth interval on adverse perinatal outcomes in Asia-Pacific region: A systematic review and meta-analysis. *PLoS ONE* 2024; 19(7): e0307942.

71. Islam MZ, Billah A, Islam MM, Rahman M, Khan N. Negative effects of short birth interval on child mortality in low- and middle-income countries: A systematic review and meta-analysis. *Journal of global health* 2022; 12: 04070.

72. He W, Jin Y, Zhu H, Zheng Y, Qian J. Effect of Chlamydia trachomatis on adverse pregnancy outcomes: a meta-analysis. *Archives of Gynecology and Obstetrics* 2020; 302(3): 553-67.

73. Helmyati S, Wigati M, Hariawan MH, et al. Predictors of Poor Neonatal Outcomes among Pregnant Women in Indonesia: A Systematic Review and Meta-Analysis. *Nutrients* 2022; 14(18): 3740.

74. Hulse GK, Milne E, English DR, Holman CDJ. Assessing the relationship between maternal opiate use and neonatal mortality. *Addiction* 1998; 93(7): 1033-42.

75. Huo N, Zhang K, Wang L, et al. Association of maternal body mass index with risk of infant mortality: a dose-response meta-analysis. *Frontiers in Pediatrics* 2021; 9: 650413.

76. Jahan S. Poverty and infant mortality in the Eastern Mediterranean region: a meta-analysis. *Journal of Epidemiology & Community Health* 2008; 62(8): 745-51.

77. Jansen LAW, Nijsten K, Limpens J, et al. Perinatal outcomes of infants born to mothers with hyperemesis gravidarum: A systematic review and meta-analysis. *European Journal of Obstetrics, Gynecology, & Reproductive Biology* 2023; 284: 30-51.

78. Jung J, Rahman MM, Rahman MS, et al. Effects of hemoglobin levels during pregnancy on adverse maternal and infant outcomes: a systematic review and meta-analysis. *Annals of the New York Academy of Sciences* 2019; 1450(1): 69-82.

79. Karami B, Abbasi M, Tajvar M. Determinants of Neonatal, Infant and Child Mortalities in Iran: A Systematic Review. *Iranian Journal of Public Health* 2024; 53(1): 104-15.

80. Keag OE, Norman JE, Stock SJ. Long-term risks and benefits associated with cesarean delivery for mother, baby, and subsequent pregnancies: Systematic review and meta-analysis. *PLoS Medicine* 2018; 15(1): e1002494.

81. Khan J, Vesel L, Bahl R, Martines JC. Timing of breastfeeding initiation and exclusivity of breastfeeding during the first month of life: effects on neonatal mortality and morbidity--a systematic review and meta-analysis. *Maternal and child health journal* 2015; 19(3): 468-79.

82. Kozuki N, Lee ACC, Silveira MF, et al. The associations of birth intervals with small-for-gestational-age, preterm, and neonatal and infant mortality: a meta-analysis. *BMC public health* 2013; 13(1): S3-S.

83. Lean SC, Derricott H, Jones RL, Heazell AEP. Advanced maternal age and adverse pregnancy outcomes: A systematic review and meta-analysis. *PLoS ONE* 2017; 12(10): e0186287.

84. Li F, Wang TT, Chen LT, Zhang SM, Chen LZ, Qin JB. Adverse pregnancy outcomes among mothers with hypertensive disorders in pregnancy: A <i>meta</i>-analysis of cohort studies. *Pregnancy Hypertension-an International Journal of Womens Cardiovascular Health* 2021; 24: 107-17.

85. Maraka S, Ospina NMS, O'Keeffe DT, et al. Subclinical Hypothyroidism in Pregnancy: A Systematic Review and Meta-Analysis. *Thyroid* 2016; 26(4): 580-90.

86. Mengistu TS, Turner JM, Flatley C, Fox J, Kumar S. The impact of severe maternal morbidity on perinatal outcomes in high income countries: Systematic review and meta-analysis. *Journal of Clinical Medicine* 2020; 9(7): 1-27.

87. Muglu J, Rather H, Arroyo-Manzano D, et al. Risks of stillbirth and neonatal death with advancing gestation at term: A systematic review and meta-analysis of cohort studies of 15 million pregnancies. *PLoS Medicine / Public Library of Science* 2019; 16(7): e1002838.

88. Nguyen DTN, Hughes S, Egger S, et al. Risk of childhood mortality associated with death of a mother in low-and-middle-income countries: a systematic review and meta-analysis. *BMC public health* 2019; 19: 1281.

89. Pineles BL, Hsu S, Park E, Samet JM. Systematic Review and Meta-Analyses of Perinatal Death and Maternal Exposure to Tobacco Smoke During Pregnancy. *American Journal of Epidemiology* 2016; 184(2): 87-97.

90. Quansah R, Armah FA, Essumang DK, et al. Association of arsenic with adverse pregnancy outcomes/infant mortality: a systematic review and meta-analysis. *Environmental Health Perspectives* 2015; 123(5): 412-21.

91. Rahman MM, Abe SK, Rahman MS, et al. Maternal anemia and risk of adverse birth and health outcomes in low- and middle-income countries: Systematic review and meta-analysis. *American Journal of Clinical Nutrition* 2016; 103(2): 495-504.

92. Rahman MA, Khan MN, Rahman MM. Maternal anaemia and risk of adverse obstetric and neonatal outcomes in South Asian countries: A systematic review and meta-analysis. *Public Health in Practice* 2020; 1.

93. Rathore SS, Oberoi S, Hilliard J, et al. Maternal and foetal-neonatal outcomes of dengue virus infection during pregnancy. *Tropical Medicine & International Health* 2022; 27(7): 619-29.

94. Robijn AL, Harvey SM, Jensen ME, et al. Adverse neonatal outcomes in pregnant women with asthma: An updated systematic review and meta-analysis. *International Journal of Gynaecology & Obstetrics* 2024; 166(2): 596-606.

95. Shah PS, Zao J, Al-Wassia H, Shah V. Pregnancy and Neonatal Outcomes of Aboriginal Women: A Systematic Review and Meta-Analysis. *Women's Health Issues* 2011; 21(1): 28-39.

96. Shen GF, Ge CH, Shen W, Liu YH, Huang XY. Association between hepatitis C infection during pregnancy with maternal and neonatal outcomes: a systematic review and meta-analysis. *European Review for Medical and Pharmacological Sciences* 2023; 27(8): 3475-88.

97. Smith ER, Hurt L, Chowdhury R, Sinha B, Fawzi W, Edmond KM. Delayed breastfeeding initiation and infant survival: A systematic review and meta-analysis. *PLoS ONE* 2017; 12(7): e0180722.

98. Tang Y, Islam N, Luo R, et al. Interpregnancy Weight Gain and Risks of Perinatal Death: A Systematic Review and Meta-Analysis. *Obesity Reviews* 2025.

99. Tekelab T, Chojenta C, Smith R, Loxton D. The impact of antenatal care on neonatal mortality in sub-Saharan Africa: A systematic review and meta-analysis. *PLoS ONE* 2019; 14(9).

100. Thomson K, Moffat M, Arisa O, et al. Socioeconomic inequalities and adverse pregnancy outcomes in the UK and Republic of Ireland: A systematic review and meta-analysis. *BMJ Open* 2021; 11: e042753.

101. Tiruye G, Shiferaw K, Shunu A, Sintayeu Y, Seid AM. Antenatal Care Predicts Neonatal Mortality in Eastern Africa: A Systematic Review and Meta-analysis of Observational Studies. *Journal of Neonatology* 2022; 36(1): 42-54.

102. Tiruye G, Shiferaw K. Antenatal Care Reduces Neonatal Mortality in Ethiopia: A Systematic Review and Meta-Analysis of Observational Studies. *Maternal and child health journal* 2023; 27(12): 2064-76.

103. Tolossa T, Fekadu G, Mengist B, Mulisa D, Fetensa G, Bekele D. Impact of antenatal care on neonatal mortality among neonates in Ethiopia: a systematic review and meta-analysis. *Archives of Public Health* 2020; 78(114).

104. Tura G, Fantahun M, Worku A. The effect of health facility delivery on neonatal mortality: Systematic review and meta-analysis. *BMC Pregnancy and Childbirth* 2013; 13: 18.

105. Veloso FCS, Kassar LDML, Oliveira MJC, et al. Analysis of neonatal mortality risk factors in Brazil: a systematic review and meta-analysis of observational studies. *Jornal de Pediatria* 2019; 95(5): 519-30.

106. Wedi COO, Kirtley S, Hopewell S, Corrigan R, Kennedy SH, Hemelaar J. Perinatal outcomes associated with maternal HIV infection: A systematic review and meta-analysis. *The Lancet HIV* 2016; 3(1): e33-e48.

107. Weightman AL, Morgan HE, Shepherd MA, Kitcher H, Roberts C, Dunstan FD. Social inequality and infant health in the UK: Systematic review and meta-analyses. *BMJ Open* 2012; 2(3).

108. Amsalu Taye Wondemagegn ATW, Animut Alebel AA, Cheru Tesema CT, Worku Abie WA. The effect of antenatal care follow-up on neonatal health outcomes: a systematic review and meta-analysis. *Public Health Reviews* 2018; 39(33).

109. Wulandari F, Budihastuti UR, Pamungkasari EP. Meta-analysis the effect of maternal obesity on the risk of premature birth and neonatal death. *Journal of Maternal and Child Health* 2021; 6(6): 719-32.

110. Xie WH, Huang H, Ji LL, Zhang ZL. Maternal and neonatal outcomes in pregnant women with psoriasis and psoriatic arthritis: a systematic review and meta-analysis. *Rheumatology* 2021; 60(9): 4018-28.

111. Yang J, D’Souza R, Kharrat A, Fell DB, Snelgrove JW, Shah PS. COVID‐19 pandemic and population‐level pregnancy and neonatal outcomes in general population: A living systematic review and meta‐analysis (Update# 2: November 20, 2021). *Acta obstetricia et gynecologica Scandinavica* 2022; 101(3): 273-92.

112. Bidhendi Yarandi R, Vaismoradi M, Panahi MH, Gåre Kymre I, Behboudi-Gandevani S. Mild gestational diabetes and adverse pregnancy outcome: a systemic review and meta-analysis. *Frontiers in medicine* 2021; 8: 699412.

113. Young MF, Oaks BM, Rogers HP, et al. Maternal low and high hemoglobin concentrations and associations with adverse maternal and infant health outcomes: an updated global systematic review and meta-analysis. *BMC Pregnancy and Childbirth* 2023; 23: 264.

114. Younger A, Alkon A, Harknett K, Louis RJ, Thompson LM. Adverse birth outcomes associated with household air pollution from unclean cooking fuels in low- and middle-income countries: A systematic review. *Environmental Research* 2022; 204.

115. Yu L, Zeng XL, Cheng ML, et al. Quantitative assessment of the effect of pre-gestational diabetes and risk of adverse maternal, perinatal and neonatal outcomes. *Oncotarget* 2017; 8(37): 61048-56.

116. Weightman AL, Morgan HE, Shepherd MA, Kitcher H, Roberts C, Dunstan FD. Social inequality and infant health in the UK: Systematic review and meta-analyses.

117. Nguyen DTN, Hughes S, Egger S, et al. Risk of childhood mortality associated with death of a mother in low-and-middle-income countries: a systematic review and meta-analysis. *BMC public health* 2019; 19: 1-21.

118. Brocklehurst P, French R. The association between maternal HIV infection and perinatal outcome: A systematic review of the literature and meta-analysis. *British Journal of Obstetrics and Gynaecology* 1998; 105(8): 836-48.

119. Pieper D, Antoine S-L, Mathes T, Neugebauer EA, Eikermann M. Systematic review finds overlapping reviews were not mentioned in every other overview. *Journal of clinical epidemiology* 2014; 67(4): 368-75.
